# Comparative immunological landscape between pre- and early-stage LUAD manifested as ground-glass nodules revealed by scRNA and scTCR integrated analysis

**DOI:** 10.1186/s12964-023-01322-x

**Published:** 2023-11-13

**Authors:** Ziqi Wang, Li Yang, Wenqiang Wang, Huanhuan Zhou, Juan Chen, Zeheng Ma, Xiaoyan Wang, Quncheng Zhang, Haiyang Liu, Chao Zhou, Zhiping Guo, Xiaoju Zhang

**Affiliations:** 1 Department of Respiratory and Critical Care Medicine, Zhengzhou University People’s Hospital, Henan Provincial People’s Hospital, Weiwu Road No.7, Zhengzhou, 450003 Henan China; 2 Department of Biochemistry and Molecular Biology, School of Basic Medicine, Tongji Medical College, Huazhong University of Science and Technology, Wuhan, Hubei China; 3 Cell Architecture Research Center, Huazhong University of Science and Technology, Wuhan, Hubei China; 4 Department of Thoracic Surgery Department, Zhengzhou University People’s Hospital, Henan Provincial People’s Hospital, Weiwu Road No.7, Zhengzhou, 450003 Henan China; 5 Department of Pathological Department, Zhengzhou University People’s Hospital, Henan Provincial People’s Hospital, Weiwu Road No.7, Zhengzhou, 450003 Henan China; 6 Zhengzhou University People’s Hospital, Henan Provincial People’s Hospital, Weiwu Road No.7, Zhengzhou, 450003 Henan China; 7 Henan Provincial Key Laboratory of Chronic Diseases and Health Management, Zhengzhou, 450003 Henan China

**Keywords:** Ground glass nodule, Lung adenocarcinoma, scRNA-seq, ScTCR-seq, Tumor microenvironment

## Abstract

**Background:**

Mechanism underlying the malignant progression of precancer to early-stage lung adenocarcinoma (LUAD) as well as their indolence nature remains elusive.

**Methods:**

Single-cell RNA sequencing (scRNA) with simultaneous T cell receptor (TCR) sequencing on 5 normal lung tissues, 3 precancerous and 4 early-stage LUAD manifested as pulmonary ground-glass nodules (GGNs) were performed.

**Results:**

Through this integrated analysis, we have delineated five key modules that drive the malignant progression of early-stage LUAD in a disease stage-dependent manner. These modules are related to cell proliferation and metabolism, immune response, mitochondria, cilia, and cell adhesion. We also find that the tumor micro-environment (TME) of early-stage LUAD manifested as GGN are featured with regulatory T (Tregs) cells accumulation with three possible origins, and loss-functional state (decreased clonal expansion and cytotoxicity) of CD8 + T cells. Instead of exhaustion, the CD8 + T cells are featured with a shift to memory phenotype, which is significantly different from the late stage LUAD. Furthermore, we have identified monocyte-derived macrophages that undergo a lipid-phenotype transition and may contribute to the suppressive TME. Intense interaction between stromal cells, myeloid cells including lipid associated macrophages and LAMP3 + DCs, and lymphocytes were also characterized.

**Conclusions:**

Our work provides new insight into the molecular and cellular mechanism underlying malignant progression of LUAD manifested as GGN, and pave way for novel immunotherapies for GGN.

Video Abstract

**Supplementary Information:**

The online version contains supplementary material available at 10.1186/s12964-023-01322-x.

## Background

The National Lung Screening Trial (NLST), one of the largest lung cancer screening trails, revealed a survival benefit of 20% reduction in mortality with low dose computed tomography (LDCT) screening compared to chest radiography (CXR) [1]. The widespread adoption of LDCT has led to an increase in the incidence of pulmonary nodules, which poses a major challenge in terms of accurate differential diagnosis and appropriate treatment. Among these pulmonary nodules, ground-glass nodule (GGN) constitute a distinct subset of the characterized by an indolent clinical course, with many of them representing precancerous or early-stage lung adenocarcinoma [2]. The continuum of malignant progression of early-stage lung adenocarcinoma including atypical adenomatous hyperplasia (AAH), adenocarcinoma in situ (AIS), microinvasive adenocarcinoma (MIA) and early invasive adenocarcinoma (IAC) [3]. AAH, AIS, MIA are considered to have excellent prognosis, and deteriorated for IAC [4]. Currently, a significant gap exists between our current practice and optimal strategy for the monitoring and intervention of GGN. This gap arises from the absence of efficient biomarkers for predicting disease behavior and the limited options beyond surgery. Meanwhile Surgery carries the potential risks of overdiagnosis and overtreatment due to the high incidence and slow growth pattern of GGNs. This predicament is primarily attributed to our incomplete understanding of the genetic and immunological dynamics, as well as the mechanisms governing the long-term indolence and factors associated with the progression of early-stage LUAD.

Cancer genesis and evolution are characterized by both the turbulence of genomic stability and immune competence [5], and they show significant divergency in the different phases [6–9]. Several studies have illustrated the genetic and mutational landscape of GGN or various phases of early-stage LUAD [10, 11]. However, there is still a need for a more comprehensive understanding of the molecular and cellular heterogeneity during the malignant progression of early-stage LUAD. Emerging scRNA sequencing technology provides a powerful tool that is more effective and accurate compared to the traditionally used bulk RNA sequencing and deconvolution estimation methods for studying of the tumor immune microenvironment [12]. In this study, we conducted droplet-based 5′ scRNA-seq and paired T-cell receptor sequencing (scTCR-seq) on the resected AIS and IAC samples presenting as GGN, along with matched normal samples. Our goal is to construct a single cell atlas and unravel the potential mechanism underlying the progression of early-stage LUAD.

## Methods

### Ethics statement, patient cohort and sample preparation

This study received approval from the Institutional Review Board (IRB) of Henan Provincial People’s Hospital (No. 201925), and all participants provided written informed consent. Patients with indeterminate pulmonary GGNs were admitted to Henan Provincial People’s Hospital and underwent lobectomy. Patients were enrolled based on the following criteria: 1) Detection of GGNs by CT scan; 2) Undergoing surgery with pathological confirmation of AIS or IAC; 3) No prior anti-cancer treatment; 4) No history of malignancies; 5) No history of autoimmune diseases, interstitial lung diseases, asthma, or chronic obstructive pulmonary disease; 6) Willingness to provide written informed consent. A total of 7 patients, including 3 with precancerous lesions and 4 with early-stage LUAD, were included in the study, and lesions as well as 5 normal tissues were collected from these patients. The nodules were divided into two pieces immediately after resection, with one piece designated for single-cell sequencing and the other for pathological diagnosis. Samples were carefully obtained from the middle area of the lesion, distinguishable from normal lung tissue by their typical grey appearance. Normal lung tissue was collected from an area at least 5 cm away from the edge of the nodule. The samples were transported in ice-cold H1640 (Gibco, Life Technologies) and were dissociated and suspended as single cell suspension with Epidermis Dissociation Kit (Human, 130–103-464) according to the manufacturer’s protocol, and Countstar Rigel S2 system was used for the qualification control of the suspension.

### RNA-Seq library preparation and sequencing

The Chromium single cell controller (10 × Genomics) was used to generate single-cell gel beads in the emulsion according to the manufacturer’s protocol with the single cell 5 'Library and Gel Bead Kit (10 × Genomics, 1,000,006) and Chromium Single Cell A Chip Kit (10 × Genomics, 120,236). Captured cells were lysed and the released RNA were barcoded through reverse transcription in individual GEMs. The cDNA was generated and then amplified, and quality assessed using an Agilent 4200. According to the manufacture’s introduction, Single-cell RNA-seq libraries were constructed using Single Cell 5’ Library and Gel Bead Kit, Single Cell V(D)J Enrichment Kit, Human T Cell (1,000,005) and Single Cell V(D)J Enrichment Kit for 10 × Genomics single-cell 5′ and TCR V(D)J sequencing. The libraries were finally sequenced using an Illumina Novaseq6000 sequencer with a sequencing depth of at least 100,000 reads per cell with pair-end 150 bp (PE150) reading strategy.

### scRNA-seq data analysis and integration

The 10 × Genomics Cell Ranger (3.0.1 version) pipeline was used for alignment, filtering, barcode counting, and unique molecular identifier (UMI) counting to generate feature-barcode matrix. GRCh38 reference genome was used for mapping. Output files were then imported into the Seurat (v3) R toolkit for quality control and downstream analysis. Cells meet either was excluded:1) nCount_RNA > 50,000; 2) nFeature_RNA > 6000; 3) nFeature_RNA < 200; 4) mitochondrial genes ratio > 20%.

After filtering, the SCTransform function from Seurat was used for the normalization and scale of the dataset as recommended by the developer, then “RunPCA” function was used to reduce the dimensionality of each dataset. Next, the “SelectIntegrationFeatures”, “PrepSCTIntegration”, “FindIntegrationAnchors” functions were successively used to identified high cell-to-cell varied features and “anchors” for the integration of individual datasets, finally we obtained the unbatched dataset that integrated all the distinct datasets with “IntegrateData” function, and this “batch-corrected” dataset allowed us to analyze all the cells together without the affection of potential batch effects. “RunPCA” was then implement again on the integrated dataset, and we clustered cells using the “FindNeighbors” and “FindClusters” functions and performed nonlinear dimensional reduction with the “RunTSNE”. The resolution was set to 0.2 in the first round of clustering to identify major cell types, and each major cell type was re-clustered using the above-mentioned pipeline with a larger resolution value.

### Marker genes identification and cell clusters annotation

After clustering of the cells, we identified mostly differential expressed genes of certain cluster compared to the rest of the clusters with the “FindAllMarkers” function of “Seurat” to identify markers of a cluster, and the cluster was annotated manually based on the markers. All the cells with two or more canonical markers or no canonical marker expressed were excluded from the subsequent analysis.

### Identification of malignant cells from normal epithelial cells

To separate malignant tumor cells from non-malignant epithelial cells, large-scale chromosomal copy number variations were inferred from the expression intensity of genes across positions of the genome by “infercnv” (v1.8.1) package with default parameters, all the epithelial cells from AIS and IAC tissues were input, all the immune cells and stromal cells was used as reference “normal” cells. CNV score was calculated as previously described, briefly, the input cells were sorted by the mean square of all the CNV values across the genome, the Pearson correlation coefficient of individual input cell’ CNV value and mean CNV value of top 5% of the cells was defined as CNV score of each cell. CNV score > 0.3 was considered as “malignant”.

### Single-Cell Weighted Gene Co-expression Network Analysis (scWGCNA)

We conducted Single-Cell WGCNA on the normal epithelial cells and inferred malignant cells to identify functional modules that are significantly relevant to developmental stages. Firstly, we identified all the genes with an expression pattern associated with developmental stages using a linear model with mixed effects by “nlme” package (v3.1–161), all the genes with an FDR < 0.001 were considered significantly associated with stages and were kept for the subsequent analysis. Then “scWGCNA” (v0.0.0.9000) package was used to construct metacells as recommended by the developer. Then an expression matrix of the metacells and genes filtered from linear model was input in to the “WGCNA” package pipeline, codes were adapted from https://horvath.genetics.ucla.edu/html/CoexpressionNetwork/Rpackages/WGCNA/index.html.

### Differential Expressed Genes (DEG) Analysis and Gene Enrichment analysis

DEG Analysis was carried out using the “FindMarker” function of “Seurat” package with default settings. Gene Ontology (GO) enrichment analysis were carried out with “clusterProfiler” R package (v4.0.5) with default settings. GSVA was also performed, 50 hallmark pathways gene-set was downloaded from Molecular Signatures Database v7.2 (https://www.gsea-msigdb.org/gsea/index.jsp). GSVA were performed with “GSVA” R package (v1.42.0), GSVA scores were compared between different groups with “limma” package (v3.48.3), an adjusted *p* value < 0.01 was considered significant.

### Definition of exhaustion, naive, cytotoxicity scores

Three well defined gene-sets were used to represent the state of cells. Exhaustion gene-set including: LAG3, TIGIT, PDCD1, CTLA4, HAVCR2. Naïve gene-set including: CCR7, TCF7, LEF1, SELL. Cytotoxic gene-set including: PRF1, IFNG, GNLY, NKG7, GZMB, GZMA, GZMH, KLRK1, KLRB1, KLRD1, CTSW, CST7. “AddModuleScore” function with default settings of Seurat package was used to calculate the scores of each cell based on the average expression of the gene-sets.

### Cell–Cell interaction analysis

“iTALK” R package (v0.1.0) was used to infer cell–cell interaction based on ligand-receptor relationships. All the DEG analysis in the processing of “iTALK” was set to “Wilcoxon rank sum test”. Codes were adopted from https://github.com/Coolgenome/iTALK.

### Cell development trajectory inferred by monocle

“Monocle” R package (v2.20.0) was used to create a new “Cell Data Set” the with counts data from RNA slot of the Seurat object as input. Highly variable expressed marker genes between cell clusters were identified by “FindAllMarkers” function of with the Seurat as genes used for dimension reduction with “reduceDimension” function, with “max_components” set to 2 and “method” choose as “DDRTree”. Next, “orderCells” was used to calculate the pseudotime value and decide the stage of each cell, which together encode where each cell maps to the trajectory.

### Translational Factors (TFs) analysis by SCENIC

“SCENIC” R package (v1.2.4) was used to investigate the translational factors (TFs) with default setting. hg19-500 bp-upstream-7species databases was downloaded from https://resources.aertslab.org/cistarget/databases for “RcisTarget”, “GRNboost”, and “AUCell”. The normalized expression matrix from Seurat object was used as input.

### TCGA data analysis

The survival analysis and gene expression correlation analysis of TCGA dataset was conducted with the on-line tool in Gepia2 developed by Zhang Zemin’s lab (http://gepia2.cancer-pku.cn/#index).

### TCR data analysis

After acquiring the raw data, Cell Ranger (v.3.0.2) was used for demultiplexing, gene quantification and TCR/BCR clonotype assignment, with GRCh38 as reference. Each unique TCR α-chain (TRA)—TCR β-chain (TRB) pair was defined as a clonotype, and only cells with one and only one clonotype were kept for further analysis. Clonal cells were defined as cells that harboring same clonotype, the number of cells with a certain clonotype indicated the degree of clonality of the clonotype.

### Immunofluorescence staining

In tissue section preparation, paraffin-embedded samples underwent deparaffinization and rehydration through a series of steps involving environmentally friendly dewaxing solutions, anhydrous ethanol, and distilled water. Antigen retrieval was performed based on specified conditions, ensuring adequate buffer presence. Subsequently, sections were subjected to hydrogen peroxide sealing and serum blocking, with primary antibodies added and incubated overnight. After washing, corresponding HRP-conjugated secondary antibodies were applied, followed by TSA dye and further washing. Microwave treatment facilitated antigen retrieval. The secondary antibodies were added and incubated overnight, followed by incubation with fluorescent-conjugated secondary antibodies. DAPI was used for nuclear counterstaining, and endogenous fluorescence was quenched if needed. Finally, slides were sealed, and images were acquired under appropriate excitation and emission wavelengths.

Primary antibody used: CD68 (Servicebio, NO: GB113150, 1:2000), FABP3 (Proteintec, NO:10,676–1-AP), FABP4 (Servicebio, NO: GB115466, 1:200). Secondary antibody: HRP-labeled Goat anti-Rabbit IgG (Servicebio, NO: GB23303, 1:500 dilution), Alexa Fluor 488-labeled Goat anti-Rabbit IgG (Servicebio, NO: GB25303, 1:400 dilution).

### Statistical analysis and plotting

The statistical methods used for each analysis are described in the above “Methods” sections and in the figure legends. In addition to above mentioned packages, “ggplot2” (v3.3.5), “RColorBrewer” (v1.1–2), “pheatmap” (v1.0.12) R packages are also used for plotting.

## Results

### Single-cell transcriptome atlas of initial and early-stage LUAD manifested as ground glass nodules

**Fig. 1:**
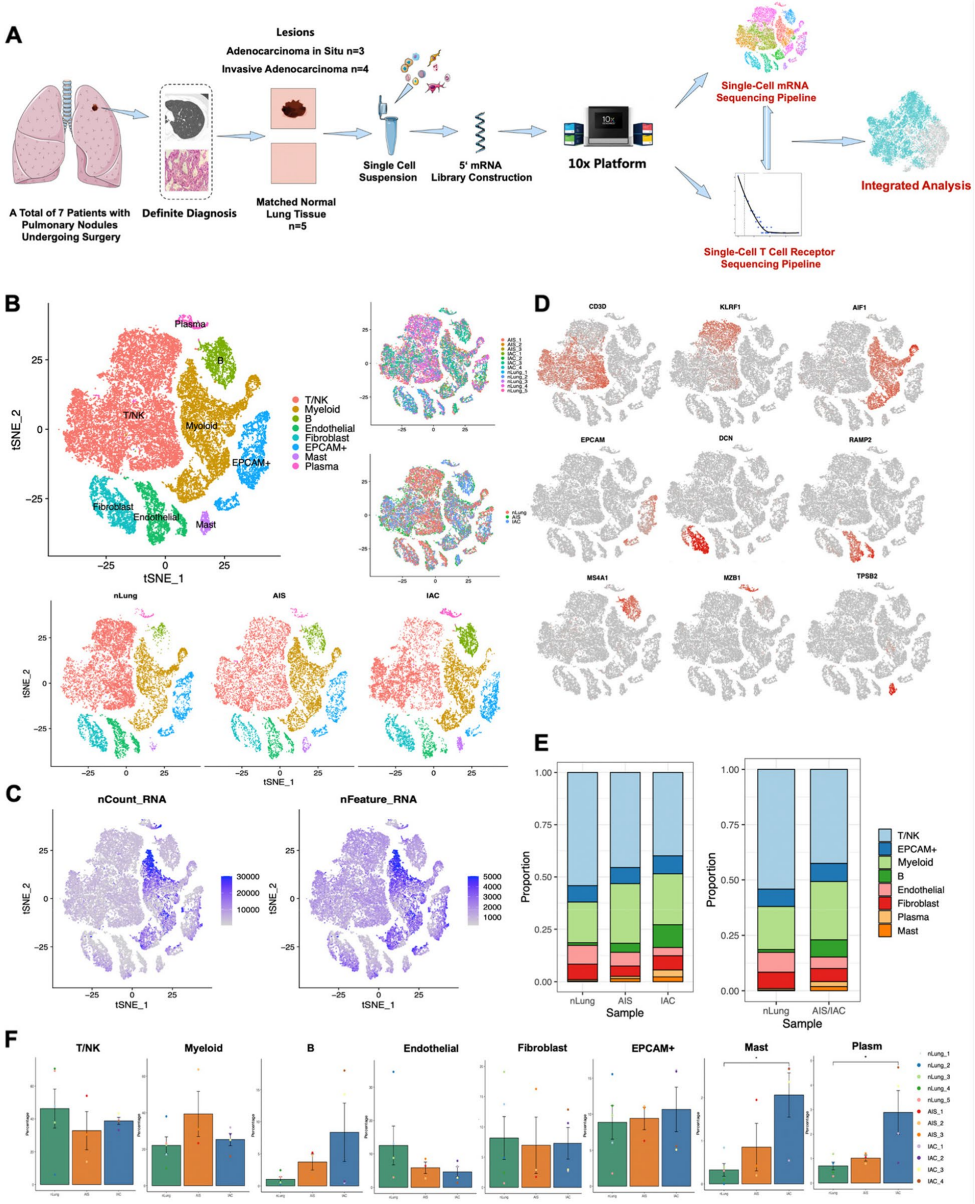
Overview of cells in major clusters. **A** Flow chart of the study design. **B** TSNE plot of 38,814 cells, colored according to cell types (Top left), according to origin of the cells (Topright),andsplitbytheoriginofthecellsrespectively (Bottom). Each dot represents a single cell. **C** TSNE plot of 38,814 cells colored by number of UMIs RNA and genes detected. Each dot represents a cell. **D** Canonical markers expression across major cellclusters. **E** The proportion of cellular composition in different patients’ groups. **F** Percentage of each cell type in different groups. Error bars represent mean ± SEM. Colored dots represent different samples. Differences with *p* < 0.05 were indicated; two-sided unpaired Wilcoxon rank sum test was used for comparison. Abbreviation: TSNE: T-distributed Stochastic Neighbor Embedding; SEM: Standarderrorofmean; UMI: UniqueMolecularIdentifier; * *p* < 0.05

A total of 12 samples, including 3 AIS, 4 IAC and 5 normal lung samples were obtained from 7 patients. These samples were subsequently digested into single cell suspensions. Then droplet-based 5′ scRNA-seq and paired T-cell receptor sequencing (scTCR-seq) was simultaneously carried out on 10X platform. Following stringent quality control measures, we successfully obtained a total of 38,814 cells (average 3,234 cells per sample) contained ~ 2.1 × 1e8 unique transcripts (average 1,671 genes per sample) for further analysis (Fig. 1A). We firstly clustered the cells into 9 types which were annotated based-on canonical markers (Fig. 1B), including CD3D + T cells, KLRF1 + NK cells, AIF1 + myeloid cells, EPCAM + cells, DCN + fibroblasts, MS4A1 + B cells, MZB1 + plasma cells, RAMP2 + endothelium cells and TPSB2 + mast cells. The cells clustered based on their types rather than the origins and the number of UMI or genes they contained (Figs. 1B, C and S1A, B, C). In addition, the markers were almost exclusively expressed in the corresponding clusters (Fig. 1D). These observations collectively suggest the successful integration of multiple datasets and the effectiveness of quality filtering. The proportion of different cell types vary across different stages, yet the differences were not statistical except for the mast cells and the plasma cells (Figs. 1E and S2). This could be attributed to the relatively small sample size, and further investigation of the data was needed.

### Different biological processes drive the malignant progression in a disease stage-dependent manner

**Fig. 2:**
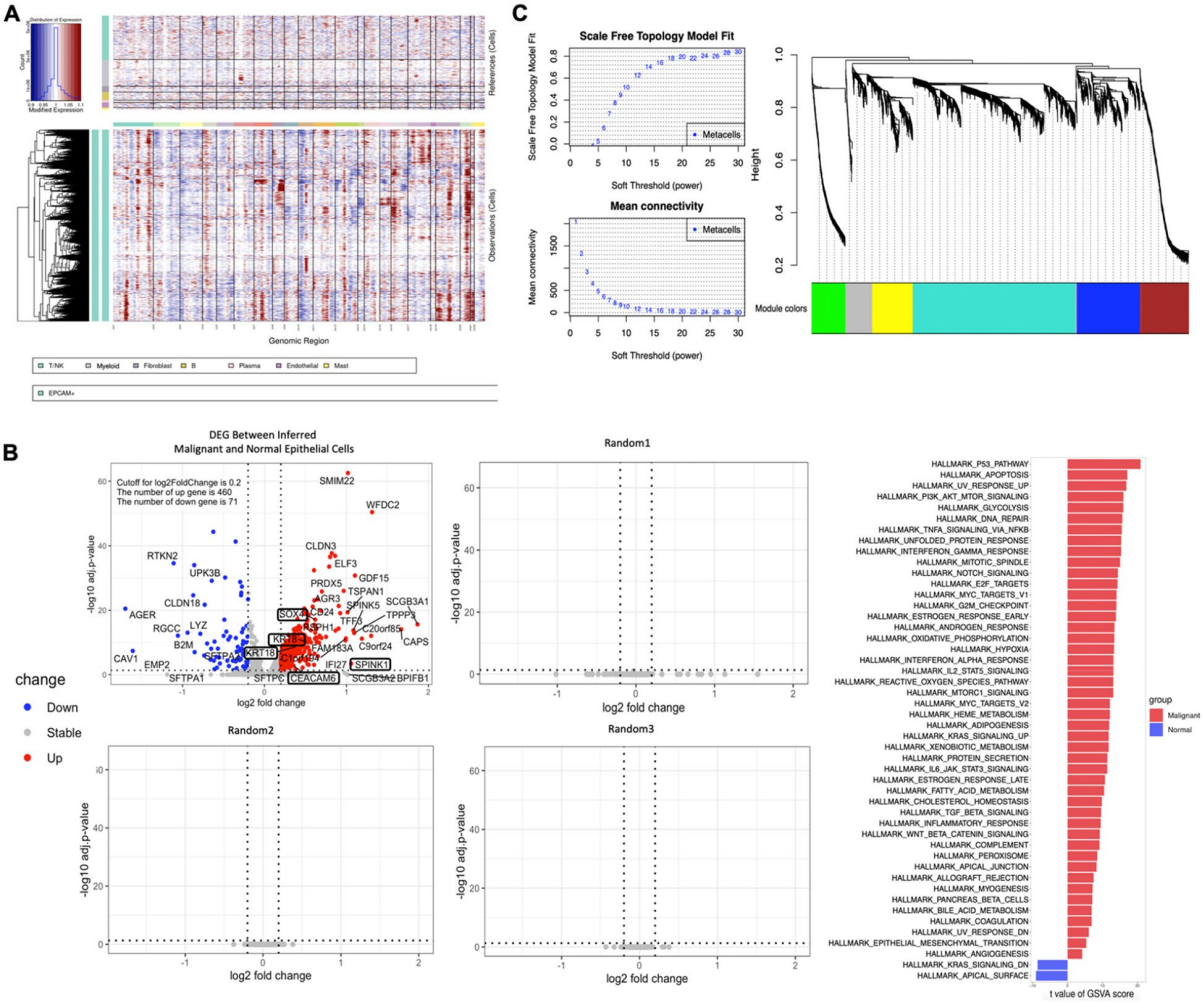
Identification of malignant cells in GGN by InferCNV and characterization of epithelia from different stage by WGCNA. **A** Heatmap showing large-scale CNVs for individual cells (rows) for all the EPCAM + cells as input, immune and stromal cells were as references (top), and large-scale CNVs were observed in malignant cells (bottom), with the color shows the log 2CNV ratio. Red represents gene amplifications and blue represents deletions. **B** Validation of inferCNV results. DEG analysis on the malignant and normal cells we inferred in the EPCAM + cells from AIS and IAC samples, and same cells randomly spited into to subsets for three times, with genes of interested was framed with black box (Left). Significantly enriched Hallmark pathways in inferred normal and malignant cells as determined by GSVA score (Right) **C** Scale-free topology model fitting, with the horizontal axis representing different soft thresholds and the vertical axis representing the value of the model (Left top); Average degree of connectivity, with the horizontal axis representing different soft thresholds and the vertical axis representing the average degree of connectivity (Left bottom). Clustering of different gene modules (Right middle). Abbreviation: AIS: Adenocarcinoma in situ; CNV: Copy Number Variation; DEG: Differential expressed gene; GSVA: Gene set variation analysis; IAC: Invasive adenocarcinoma; WGCNA: Weighted correlation network analysis

Next, we carried out sub-clustering and explored the characteristics of each cell type. Firstly, we focused on the EPCAM + cells. A total of 3,122 EPCAM + cells were identified, with 1,183 originating from normal tissues (37.9%), 866 from AIS (27.7%), and 1,073 from IAC (34.4%). Cells from normal tissues were subclustered and annotated as AT1 cells, AT2 cells, club cells and ciliated cells (Figs. S3A, B), which were similar to previous studies [13]. For the EPCAM + cells from AIS and IAC, inferCNV was carried out to identify malignant cells with all the immune and stromal cells set as reference (Fig. 2A). CNVscore was defined as previously reported (Experimental Section), and finally 1,528 malignant cells (78.8%) were identified from 1,939 EPCAM + cells (CNVscore > 0.3). To confirm the result given by inferCNV, we carried out DEG analysis on the inferred malignant cell and inferred normal cells from AIS and IAC, revealed 460 upregulated genes and 71 downregulated genes (Fig. 2B). Among the upregulated genes we noticed markers those were associated with cancer genesis and development, including SOX4, SPINK1, CEACAM6, KRT8 and KRT18 [14–16]. Contrarily, randomly split of the cells we input in the inferCNV identify no DEGs (Figs. 2B and S4A). Gene set variation analyses (GSVA) using the 50 Hallmark gene-sets (MSigDB) also revealed that almost all the cancer related pathways were significantly enriched in the inferred malignant cells (Fig. 2C). All these results suggested the reasonability of inferCNV’s annotation.

**Fig. 3:**
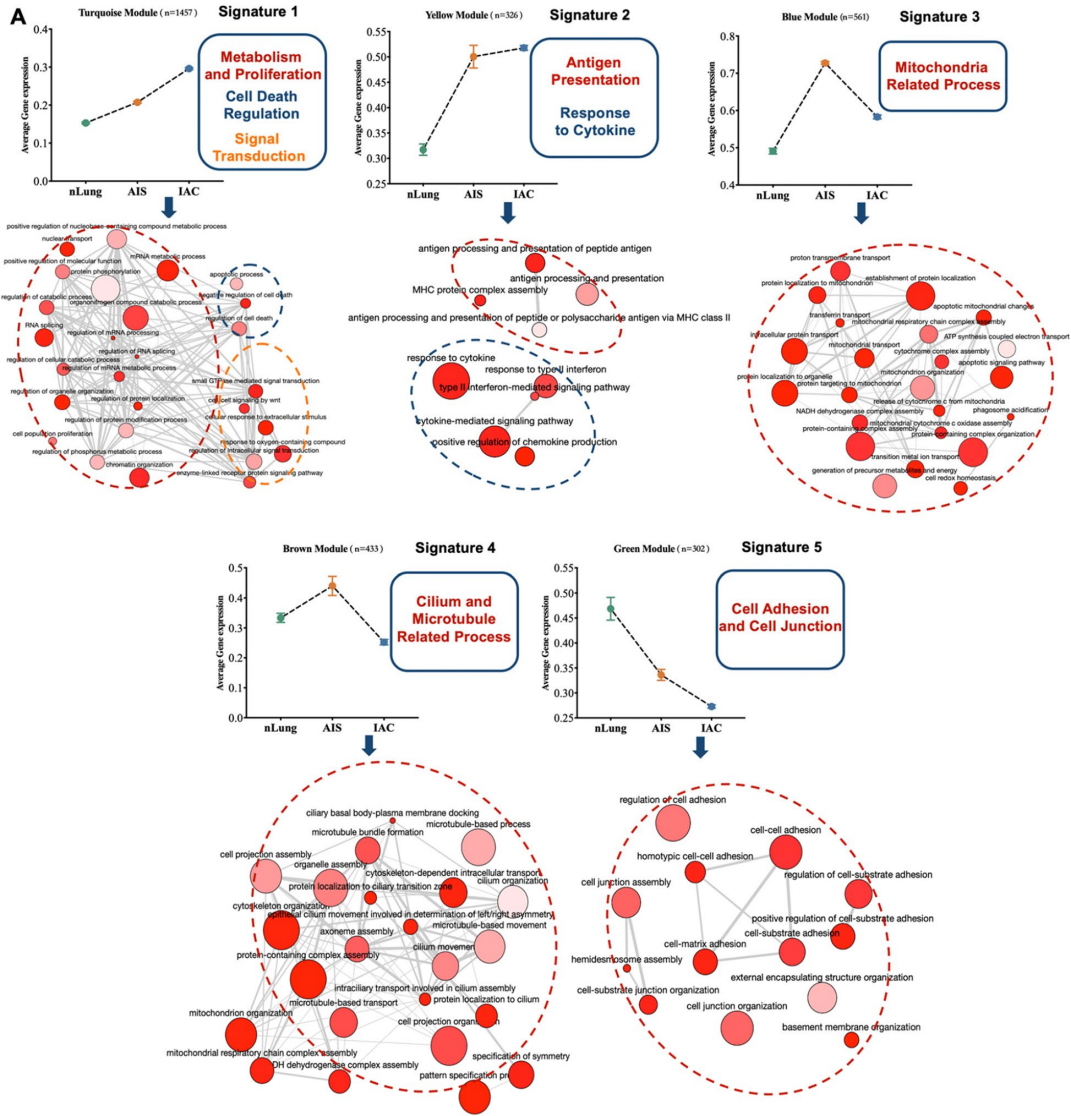
Five modules of co-expressed genes identified with linear combined with scWGCNA. **A** The gene-expression measurement represents the mean expression level of genes in each module, and error bar indicated SEM. Bubble plot beneath each gene-expression measurement was created on Revigo, shows the over-represented GOBP terms in each module, the color of the bubbles corresponds to the FDR value, the size of the bubble corresponds to the LogSize value for the GO Term, and the edge means the 3% of the strongest GO term pairwise similarities, as per to the developer of Revigo. GO terms that can be clustered into one category were circle by dotted oval with color that corresponded to the color of signature on the right top of each subfigure. Abbreviation: FDR: False discovery rate; GOBP: Gene Ontology Biological Process; SEM: Standard error of mean

Next, we considered EPCAM + cells from normal tissues (*n* = 1,183), and malignant cells inferred by inferCNV from AIS (*n* = 604) and IAC (*n* = 924) as three different stages during LUAD development, to explore the transcriptome evolutionary trajectories from normal tissue to AIS, and then IAC. We initially identified 3,369 genes with expression levels that exhibited a strong correlation with different disease stages, using a linear mixed-effects model with a false discovery rate (FDR) threshold of < 0.001. Subsequently, employing the single-cell weighted gene co-expression network analysis (scWGCNA) package [17], we separately merged the three groups of EPCAM + cells into metacells (as described in the Experimental Section). This process yielded a total of 401 metacells, with the following distribution: nLung: *n* = 179; AIS: *n* = 63; IAC: *n* = 159. The gene expression matrix of the 3,369 stage-associated genes within these 401 metacells was subjected to subsequent analysis. Ultimately, we identified co-expressed genes with distinct expression patterns and organized them into five gene modules using WGCNA. These modules were color-coded as turquoise, yellow, brown, blue, and green, while a grey module represented genes that could not be assigned to any other modules (Fig. 3A).

The turquoise module (1,457/3,369, 43.2%) contained the majority of the genes, followed by blue module (561/3,369, 16.7%), the brown module (433/3,369, 12.9%), yellow module (362/3,369, 10.7%), green module (302/3,369, 9.0%), and grey module, which had the fewest genes (254/3,369, 7.5%) (Figs. 3A, B). Among these modules, turquoise and yellow represented ascending types, with the turquoise module showing a linear progression from normal tissue to cancer, and yellow module significantly rosed from AIS stage and maintaining high expression levels in the IAC, possibly representing early-phase alterations in cancer cells in response to immune stimulation. The blue and brown modules represented biphasic gene-expression module, which means that the genes within them reached a peak of expression in AIS. Additionally, the green module represents the descending module (Figs. 3A, B).

We summarize signatures of each module through functional enrichment analysis with redundancy reduction by Revigo [18] to the pathways (Fig. 3B). The turquoise module was primarily associated with metabolism and proliferation, cell death regulation and signal transduction, supporting the requirements for exponential growth and proliferation in the process of acquiring malignant phenotypes (Figs. 3B and S5A). The yellow module was strongly linked to antigen presentation and response to cytokine, representing interactions between hyperplasia or cancerous cells and the immune system, which together reflecting a stimulated immune response from the precancer stage of LUAD (Figs. 3B and S6A). In addition, the blue module was over-represented by pathways related to mitochondria, including the respiratory chain, cytochrome complexes and oxidative phosphorylation, suggesting unique energy metabolism reprogramming in the transition of pre-malignancy to cancerous cells (Figs. 3B and S7A). The brown module was associated with cilium and microtubule processes (Figs. 3B and S8A). Lastly, the green module, which exhibited decreased expression with disease progression, was enriched in cellular adhesion related pathways, possibly indicating the process of acquiring the ability to detach from extracellular matrix and invade (Figs. 3B and S9A).

The network between genes with the highest eigengene-based connectivity (kME value) in each module were also visualized (Fig. S10A). These findings suggest that the development of LUAD is a signaling-intense and metabolically demanding process with high dynamic and complexity, along with the evoking of immune response and loss of detachment in the initial stage of LUAD.

### Accumulation of tregs with diverse sources and loss-functional State of CD8 + T cells instead of exhaustion features the TME of initial and early-stage LUAD manifested as GGN

**Fig. 4:**
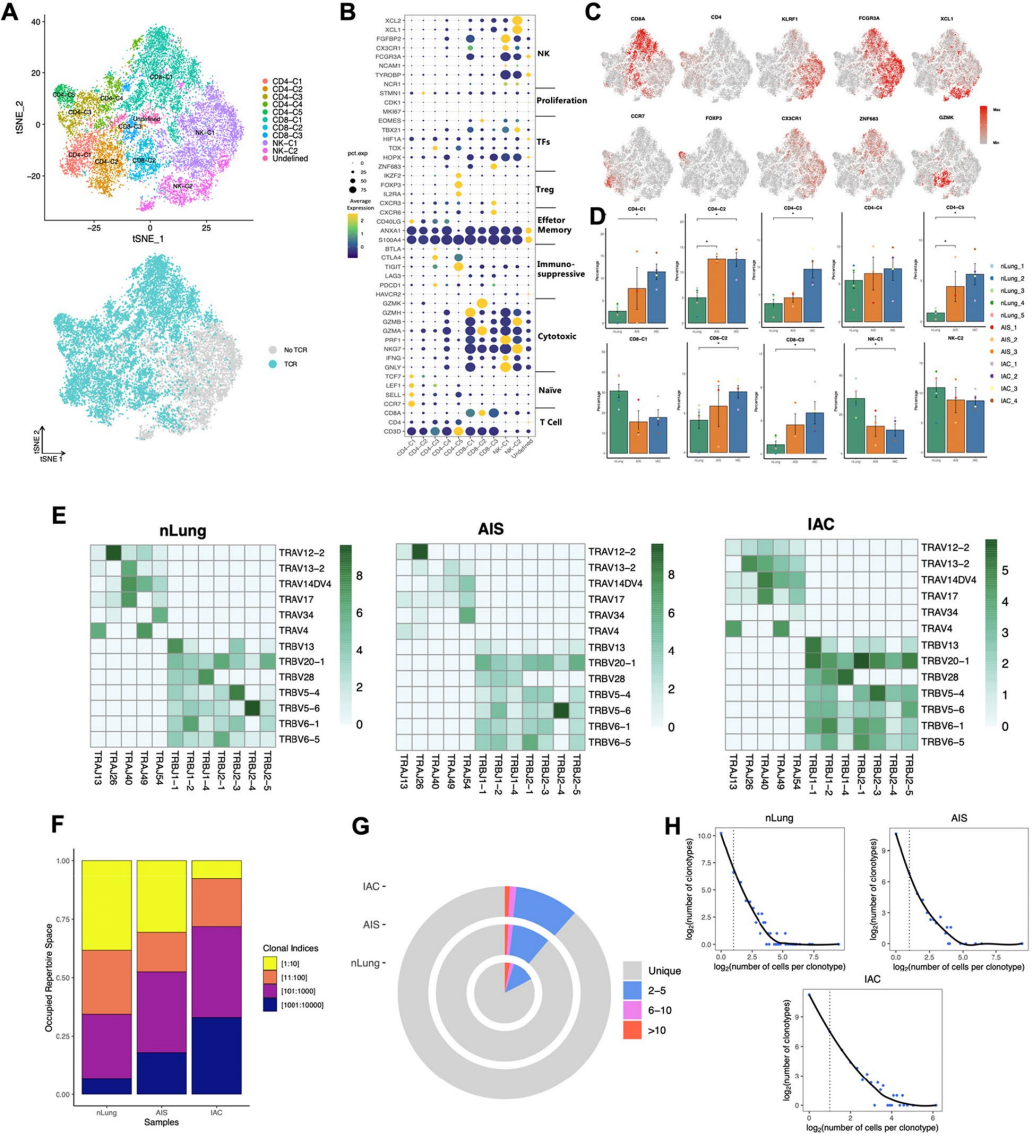
Recluster of T/NK cells and overview of TCR distribution across different groups. A. TSNE plot of 11,847 T/NK cells reclustered, colored according to cell subtypes (Top), colored according to TCR detected (Bottom). Each dot represents a single cell. B. Dot plot of functional genes expression in each T/NK subclusters. C. Canonical markers expression for each T/NK subclusters. D. Percentage of each T/NK subtypes in different groups. Error bars represent mean ± SEM. Colored dots represent different samples. Differences with *p* < 0.05 were indicated; two-sided unpaired Wilcoxon rank sum test was used for comparison. E. LOESS fitting plot showed the correlation between the number of T cell clones and the number of cells per clonotype. Solid line is used to display the correlation, and dashed line is used to separate between nonclonal and clonal cells. F. Status of immune repertoire space occupancy of clones across different groups by stacked bar chart. The occupancy of the clone space was analyzed by sorting all clonotypes in descending order based on their frequency of occurrence. The data were then separated into different bins using cutoff values of 10, 100, 1000, and 10,000, and stacked bar chart was then plotted to visualize the distribution of clonotypes among the different bins. G. The percentage of clones with different frequencies across different groups. H. Status of TRA/B genes usage across different groups. The colors indicate the usage percentage of specific V-J gene pairs. Abbreviation: LOESS: Locally Weighted Scatterplot Smoothing; SEM: Standard error of mean; TCR: T cell repertoire

T cells and NK cells are closely related cell components and serve as the major effector cells in the TME [19]. Subclustering of a total of 18,268 T/NK cells revealed eight subtypes of T cells and two subtypes of NK cells (Figs. 4A and S11A). Among these, there were five clusters of CD4 + T cells (*CD3* + *CD4* + *CD8-*), each with distinct characteristics. CD4-C1 was defined as naïve CD4 + T cell (*CCR7* + *SELL* +), CD4-C2 as effector memory CD4 + T cell (*ANXA1* + *GZMA* +), CD4-C3 as exhausted CD4 + T cells (*PDCD1* + *TOX* + *CTLA4* + *TIGIT* +), CD4-C4 as cytotoxic CD4 + T cell (*GZMA* + *GZMH* +), and CD4-C5 as regulatory CD4 + T cell (CD4 + Treg) (*FOXP3* + *IL2RA* + *CTLA4* + *TIGIT* +) (Fig. 4B, C).

Furthermore, three subtypes of CD8 + T cells (*CD3* + *CD4-CD8* +) were identified. CD8-C1 was defined as cytotoxic CD8 + T cell (*CX3CR1* + *GZMH* + *GZMB* + *GZMA* +). CD8-C2 was characterized with low expression of coinhibitory molecules and high expression of GZMK, displaying a pattern reminiscent of previously reported pre-exhausted CD8 + T cells [20, 21], and was thus defined as GZMK + pre-Exhausted CD8 + T cell. CD8-C3 showed strong expression of ZNF683, CXCR3 and CXCR6, resembling tissue-resident memory CD8 + T cell, and was also considered as pre-exhausted CD8 + T cells as an alternative evolution pathway of naïve CD8 + T cells to terminal exhausted CD8 + T cells. Therefore, we defined CD8-C3 as ZNF683 + pre-Exhausted CD8 + T (*ZNF683* + *CD8* + Texp) (Fig. 4B, C) [21, 22]. NK cells were annotated as NK-C1 (*CD16* +) and NK-C2 (*CD16-XCL1* + *XCL2* +) based on the expression pattern of FCGR3A (which encodes CD16) (Fig. 4B, C). Notably, no terminal exhausted CD8 + T cell clusters expressing high level coinhibitory molecules were identified. Additionally, a cluster referred to as the 'Undefined cluster' displayed neither T nor NK markers expression and exhibited significantly low gene counts, and was consequently considered a cluster of low-quality cells, subsequently excluded from further analysis.

Compared to normal lung tissue, IAC exhibited a significantly increase in the proportions of several cell types, including naïve CD4 + T cells (CD4-C1, *p* = 0.016), exhausted CD4 + T cells (CD4-C3; *p* = 0.016), effector memory CD4 + T cells (CD4-C2; *p* = 0.016), CD4 + Treg (CD4-C5; *p* = 0.016), ZNF683 + pre-exhausted CD8 + T cells (CD8-C3; *p* = 0.016) and GZMK + pre-exhausted CD8 + cells (CD8-C2; *p* = 0.032). Conversely, CD16 + NK cell (NK-C1; *p* = 0.032) decreased in IAC compared to normal lung tissue. CD8-C1 also exhibited a reduction with marginal significance (*p* = 0.063). In AIS, most cell types displayed intermediate levels between those observed in normal lung tissue and IAC. Of note, effector memory CD4 + T cells (CD4-C2; *p* = 0.036) and CD4 + Treg (CD4-C5; *p* = 0.036) showed significant increase in AIS compared to normal lung tissue (Fig. 4D).

**Fig. 5:**
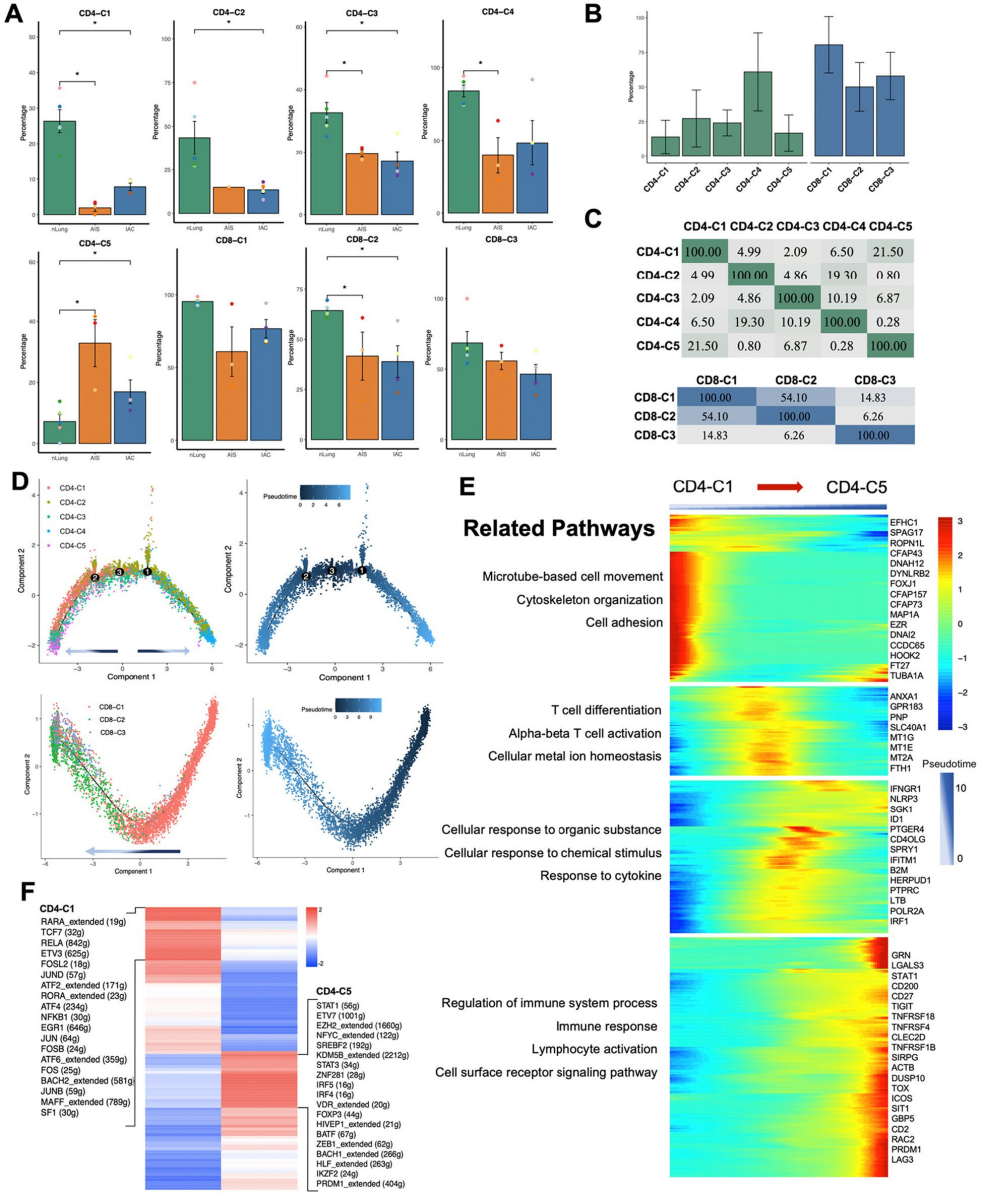
Clonal expansion analysis and development trajectory inferring based on combined analysis with Monocle2. **A** Clonal expansion status indicated by the percentage of clonal cells in each cell clusters. **B** TCR overlapping in CD4 + T cells and CD8 + T cells respectively. Color responded to the percentage of overlapping. **C** Percentage of clonal cells in each T cell subclusters across different groups. Error bars represent mean ± SEM. Colored dots represent different samples. Differences with *p* < 0.05 were indicated; two-sided unpaired Wilcoxon rank sum test was used for comparison. **D** Development trajectory of CD4 + and CD8 + T cells inferred by Monocle2, colored according to cell types (Top), and colored according to pseudotime value (Bottom). Gradient-colored arrow indicated the direction of development inferred. **E** Heatmap showing differentially expressed genes arranged in pseudo temporal patterns. Related GO terms revealed biological functions and representative genes in each cluster were indicated due to space limitation. **F** The most differentially enriched TFs between naïve CD4 + T cells and Tregs investigated by SCIENIC algorithm. Abbreviation: GO: Gene Ontology; SCIENIC: Single-cell regulatory network inference and clustering; SEM: Standard error of mean; TFs: Translational factors

Simultaneously, we retrieved TCR sequencing data from 8,451 T cells (71.33%, 8,451/11,847) that had productive and unique TCR α- and β-chain pairs after stringent quality-control filtering (Fig. 5A), with a total of 5,774 distinct clonotypes. In addition, AIS have a comparable V(D)J genes usage with nLung, which were apparently differed from IAC (Fig. 5B). Such selective usage of V(D)J genes suggests that different immunodominant epitopes may drive the molecular composition of T cell responses and may be associated with disease stage specific responses. Furthermore, AIS and IAC both displayed significantly decreased clonal expansion when compared to nLung, as evidenced by an increase in the number of unique clonal types (Figs. 5C, D) and a decrease in the percentage of clonal cells (Figs. 5E and S11B) from nLung to IAC. Additionally, CD4 + T cells generally showed lower clonal percentage compared to CD8 + T cells, with the exception of the Cytotoxic CD4 + T cell (CD4-C4) cluster (Fig. S11C). This observation suggests higher clonal activity of cells with effector function. In particular, CD4-C1, CD4-C2, CD4-C3, CD4-C4, CD8-C2 from AIS or IAC showed a significant decline in the percentage of clonal cells compared to nLung. Notably, CD4 + Treg cells(CD4-C5) were the only cluster that displayed a higher percentage of clonal cells in AIS/IAC compared to nLung (Fig. 5E). This finding is consistent with a previous study in esophageal cancer [23] and suggests that the accumulation of Treg cells in AIS and IAC is at least partly attributed to the local expansion of certain clonotypes.

**Fig. 6:**
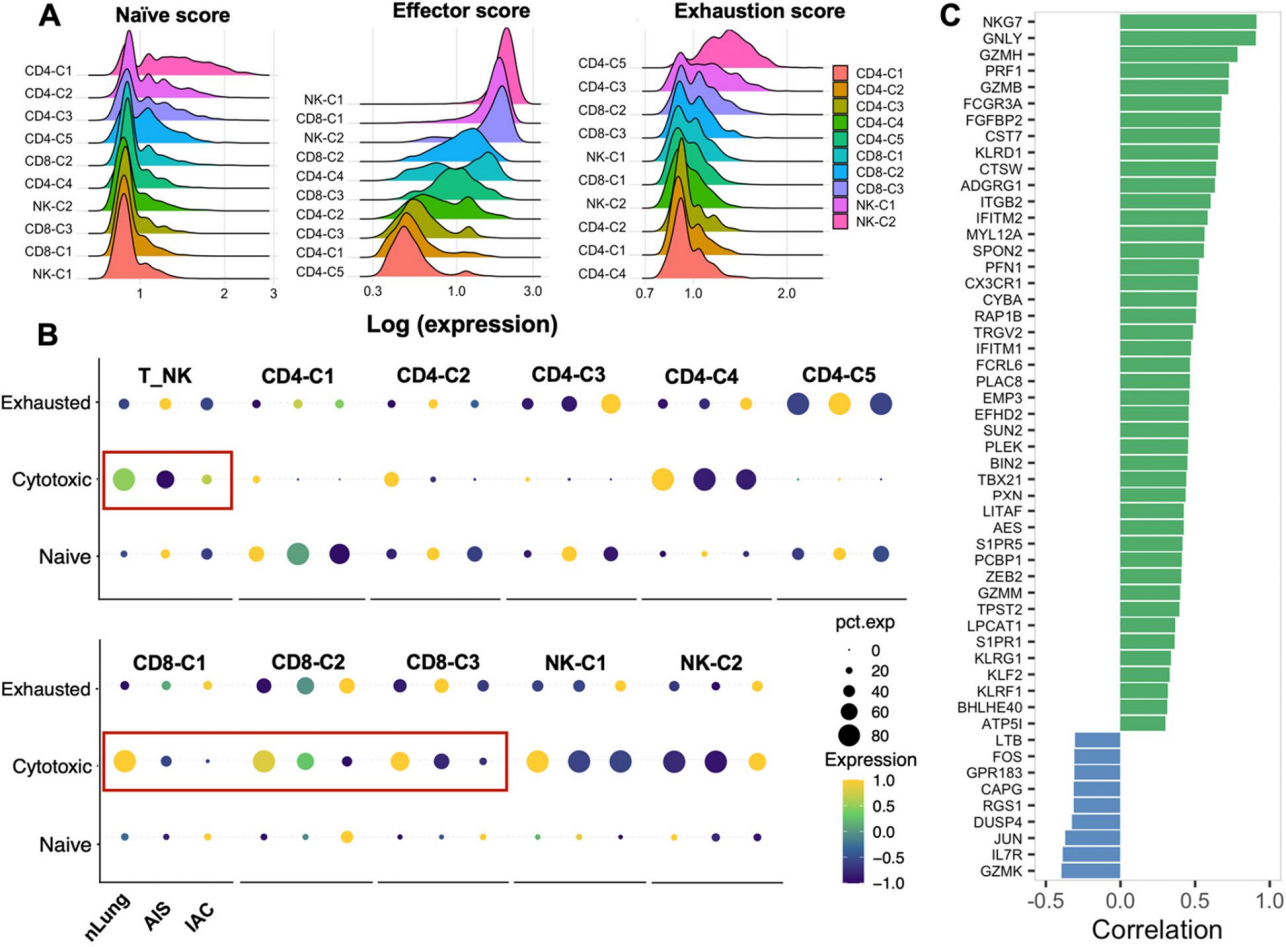
Functional characteristics of different T/NK subclusters. **A** Ridge plot showing the naïve score, effector, and exhaustion score across different T/NK subclusters, arranged in descending order from top to bottom based on the logged expression values of each score. **B** The differential expression of different functional scores across different groups. Red boxes highlight the decrease of cytotoxic score along the progression of disease. **C** Genes significantly correlated with cytotoxic score in all groups of CD8 + T cells and differentially expressed in CD8 + T cells from AIS/IAC compared with nLung. Green bars indicated genes that are positively correlated with cytotoxic score and downregulated in CD8 + T cells from AIS/IAC; Blue bars indicated genes that are negatively correlated with cytotoxic score and upregulated in CD8 + T cells from AIS/IAC; length of the bar indicated the value of Pearson correlation coefficient

We then conducted an analysis of the overlapping of TCR in different subclusters of CD4 + and CD8 + T cells to infer the lineage tracing of various T cell clusters. The results revealed that cytotoxic CD8 + T cells and GZMK + pre-exhausted CD8 + T cells shared the highest number of identical clonotypes, with an overlapping rate of 54.10%. This was followed by naïve CD4 + T cells and Treg cells, which exhibited a 21.50% overlapping rate, and effector memory CD4 + T cells and cytotoxic CD4 + T cells, which shared 19.3% of identical clonotypes (Fig. 5F). These findings indicate specific cell fate commitments. Moreover, we employed Monocle2 [24], an unsupervised method frequently used to infer the developmental trajectory of different cell types. The results of the Monocle2 analysis supported the findings of the TCR overlapping analysis. It demonstrated that naïve CD4 + T cells, effector memory CD4 + T cells, and cytotoxic CD8 + T cells occupied the root of the trajectories and preferentially developed into CD4 + Treg cells, cytotoxic CD4 + T cells, and GZMK + pre-exhausted CD8 + T cells, respectively (Fig. 6A). This observation also suggests that differentiation from naïve CD4 + T cells could be another potential source of Treg cell accumulation in the initial stage of LUAD, in addition to local expansion. Based on the inferred lineage relationship between naïve CD4 + T cells and Treg cells, we subsequently identified genes with dynamic expression over pseudotime to investigate the transcriptome transition during the development of Treg from naïve CD4 + T cells. Differential expressed genes were identified and clustered into four groups. Group 1 were primarily associated with cell adhesion and cell movement, which is consistent with the high migration capacity of naïve CD4 + T cells compared to Treg cells [25, 26]. Group 2 and Group 3 were enriched for the process related to T cell activation and cellular responses to various stimuli, reflecting the differentiation process. It's worth noting that genes associated with metal ion regulation, including zinc regulators MT1 and MT2, which have been reported to be enriched in dysfunctional CD8 + tumor-infiltrating T cells [27], and iron regulators SLC40A1 and FTH1, were also found to be involved in this process. This finding suggests that these genes could potentially serve as therapeutic targets to interfere with the differentiation into Treg cells. Additionally, Group 4 was enriched in genes related to immune system regulation (Fig. 6B). We employed the Single-cell Regulatory Network Inference and Clustering (SCIENIC) algorithm [28] to identify differentially expressed transcription factors between naïve CD4 + T cells and Treg cells. This analysis demonstrated an enrichment of the methylase EZH2 and demethylase KDM5B in Treg cells compared to CD4 + T cells (Fig. 6C). This suggests that the differentiation from naïve CD4 + T cells to Treg cells involves extensive epigenetic reprogramming [29].

Then we use three well-defined gene-sets that representing naïve score (n-score), cytoxicity score (c-score) and exhaustion score (e-score) to further characterize the functional state of each cell cluster. As expected, naïve CD4 + T cells (CD4-C1; mean n-score = 0.31) exhibited the highest naïve score, followed by effector memory CD4 + T cells (0.08); NK-C1(mean c-score = 0.99), cytotoxic CD8 + T cells (CD8-C1; 0.69) and NK-C2 (0.66) representing the most cytotoxic cell types, CD4 + Treg showed least cytotoxic effector expression. Furthermore, CD4 + Treg (CD4-C5; mean exhausted score = 0.30) and exhausted CD4 + T cells (CD-C3; 0.11) represented the most exhausted cell types (Fig. 6D), and we’ve noticed that all subclusters of CD8 + T cell experienced a dramatic decrease of cytotoxicity along with the disease progression without appearance of exhausted phenotype (Fig. 6E), based on this observation, we propose that the loss of functional state, characterized by decreased cytotoxicity without a clear transition to terminal exhaustion, may be the key feature of lymphocytes in the TME of early-stage LUAD. This is distinct from the late stage, which is characterized by the dominance of exhausted CD8 + T cells [20, 30].

To explore the shared biological circuits contributing to dysfunction in CD8 + T cells, we conducted a selection process to identify genes meeting the following criteria: 1) genes with expression pattern correlated with cytotoxic score (Pearson correlation coefficient ≥ 0.3); 2) genes significantly differentially expressed between nLung and AIS/IAC (logFC ≥ 0.3 and adj. *p* value < 0.05) (Fig. 6F). Among the upregulated genes, IL7R and LTB are indicative of a memory phenotype [21]. This aligns with previous study suggesting that continuous antigen stimulation can lead to a memory phenotype in T cells [31]. Additionally, the high expression of DUSP4 has been associated with the acquisition of a memory phenotype and CD4 + T cell senescence in patients with idiopathic CD4 lymphopenia [32] (Fig. 6C). Notably, we also observed upregulation of JUN and FOS, both components of the AP-1 complex. Interestingly, the AP-1 complex has been reported as a key transcriptional regulator of genes associated with effector responses and exhaustion. However, exhausted T lymphocytes typically exhibit low expression of AP-1. This suggests that there may be other molecules or mechanisms aside from AP-1 involved in regulating the dysfunctional state of T cells in our dataset [33]. On the other hand, 44 genes positively correlated with the cytotoxic score were downregulated in AIS and IAC. In addition to effector molecules and T cell activation markers [34] such as NKG7, GNLY, GZMH, PRF1, CCL4, we also identified molecules involved in cytoskeleton regulation and lymphocyte recruitment, including SUN2 and SPON2 [35, 36]. In addition, EFHD2, PCBP1, ADGRG1, PXN, PLAC8 have recently been reported [37–40] to be critical for the stability of effector T cell function. KLF2 and its target gene S1PR1, as well as ZEB2 and S1PR5, were significantly downregulated, consistent with the acquisition of a residential memory phenotype [41–43]. Additionally, several genes with less-known associations with T lymphocyte function, such as MYL12A, TPST2, ATP5I, LPCAT1, warrant further investigation.

### Monocyte-derived macrophage experienced lipid-phenotype transition and prime the suppressive microenvironment

**Fig. 7:**
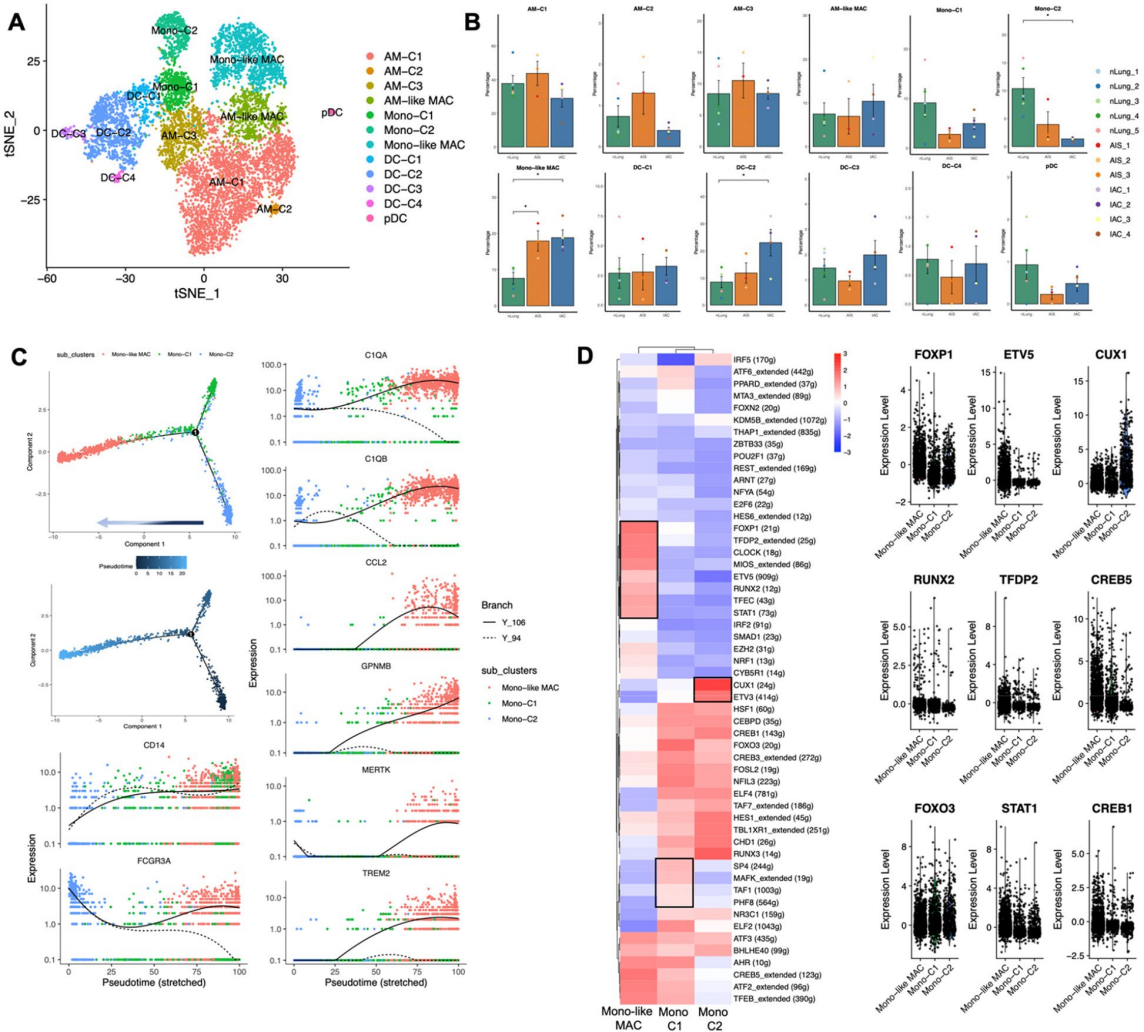
Recluster of myeloid cells and developmental trajectory of monocytes inferred by Monocle2. **A** TSNE plot of 9,179 myeloid cells reclustered, colored according to cell subtypes. **B** Percentage of each myeloid subtypes in different groups. Error bars represent mean ± SEM. Colored dots represent different samples. Differences with *p* < 0.05 were indicated; two-sided unpaired Wilcoxon rank sum test was used for comparison. **C** Developmental trajectory of monocytes and Mono-like MAC inferred by Monocle2, and the expression pattern based on the trajectory of genes of interest. **D** The most differentially enriched TFs between monocytes and Mono-like MAC investigated by SCIENIC algorithm (Left), the TFs exclusively enriched in each clustered were highlighted with black boxes. Expression of representative TFs (Right). Abbreviation: SCIENIC: Single-cell regulatory network inference and clustering; SEM: Standard error of mean; TFs: Translational factors; TSNE: T-distributed Stochastic Neighbor Embedding. * *p* < 0.05

Myeloid cells are major player in the suppressive microenvironment of tumor and was shown to be involved from the very beginning of cancer genesis [44]. Upon reclustering of 9,179 myeloid cells, we identified 12 subclusters (Figs. 7A, S12A, B). Among these, we identified 4 clusters of alveolar macrophages (AM) characterized by high expression of PPARG, FABP4 and MARCO (AM-C1 to AM-C4) (Fig. 7A, S12A, B). Notably, AM-C3 also highly expressed FCN1, a marker for monocytes, which imply its origination from monocytes. Furthermore, 2 clusters of monocytes were identified, Mono-C1 corresponding to classical monocytes (*CD14* + *FCN1* +), and Mono-C2 was annotated as non-classical monocyte (*CD16* + *FCN1* +). Moreover, a cluster of macrophages that simultaneously showed expression pattern of monocyte (*CD14* + *CD16* +) and macrophage (*CD68* + *MRC1* +) were defined as Mono-like MAC. Interestingly, this cluster of macrophages also highly expressed previously reported tumor associated macrophage (TAM) related genes, including SLC40A1, although a well-defined TAM marker SPP1 [45] was not expressed in Mono-like MAC. We also retrieved 5 clusters of dendritic cells (DCs), DC-C1 was defined as Mono-DC due to highly expressed FCN1 (*FCN1* + *CLEC10* +), DC-C2 as CD1C + DC (*CD1C* + *FCGR2B* +), DC-C3 as LAMP3 + DC (*LAMP3* +), DC-C4 as CLEC9A + DC (*CLEC9A* +), and a cluster of plasmacytoid dendritic cell (pDC, *LILRA4* +) was also identified (Figs. 7A, S12A, B). We also examined classical M1 and M2 polarization markers in myeloid cells and found no typical M1 or M2 expression patterns in any of the subclusters. This suggests that the status of myeloid cells in the tumor microenvironment is more complex than the classic M1 and M2 models [46] (Fig. S13A).

Compared to nLung, IACs were enriched for Mono-like MAC (*p* = 0.016) and were deprived of CD16 + CD14- non-classical monocytes (Mono-C2, *p* = 0.016). Notably, Mono-like MAC were also significantly enriched in AIS compared to nLung (*p* = 0.036) (Figs. 7B and S13B), suggesting a potential role in the very early stage of carcinogenesis.

**Fig. 8:**
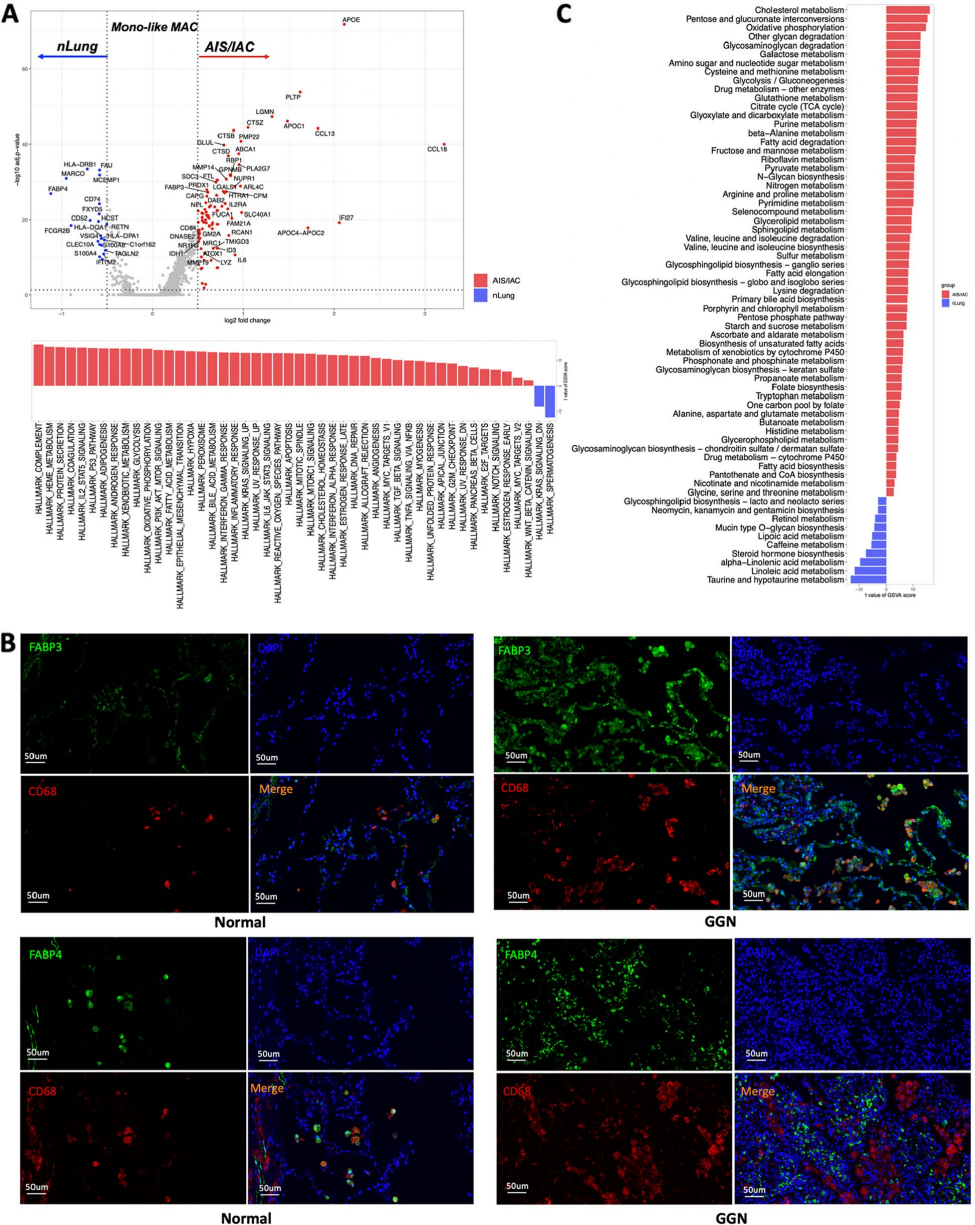
Functional analysis of Mono-like MAC subclusters. A. DEGs between Mono-like MAC isolated from nLung and AIS/IAC (Top). Significantly enriched Hallmark pathways in Mono-like MAC isolated from nLung or AIS/IAC as determined by GSVA score (Bottom). B. Significantly enriched metabolism-related pathways in Mono-like MAC isolated from nLung or AIS/IAC as determined by GSVA score. C. Representative immunofluorescence images showing FABP3 and FABP4 expression on macrophages in normal tissues and LUAD manifested as GGN. CD68 were stained red, FABP3 and FABP4 were stained green, and nuclei were stained blue (DAPI). Abbreviation: AIS: Adenocarcinoma in situ; DEG: Differential expressed gene; GSVA: Gene set variation analysis; IAC: Invasive adenocarcinoma

As previously mentioned, Mono-like MAC exhibits a high level of expression for genes related to monocytes. To explore their potential transitional relationships, we applied Monocle2 analysis to Mono-C1, Mono-C2, and Mono-like MAC. As expected, Mono-like MAC appeared at the end of the developmental branches, while Mono-C1 and Mono-C2 were located closer to the beginning (Fig. 7C). The representative genes of monocytes, macrophage and TAM also showed no random expression patter (Figs. 7D and S14A), all these suggest the monocytic origin of this macrophage cluster. Next, we applied DEG and functional enrichment analysis on the Mono-like Mac cluster. Moreover, distinct pattern of TFs that may mediate the differentiation of monocytes into Mono-like MAC were revealed by SCIENIC. Compared to monocytes, the Mono-like MAC cluster showed exclusive high levels of FOXP1, TFDP2, CLOCK, MIOS, RUNX, ETV5, TFEC, and STAT1, with ETV5 exhibiting the most variable expression and targeting the largest number of genes. This suggests its potential vital role in regulating macrophage differentiation and lipid metabolism reprogramming (Figs. 7E and S14B). In addition, comparing to their counterpart in normal lung tissue, Mono-like Mac in AIS/IAC upregulated numerous lipids metabolism and transportation related genes, including APOE, APOC1, PLTP, ABCA1, PLA2G7 etc. This indicated that this macrophage cluster undergoes significant lipid metabolism reprogramming (Fig. 8A), a phenotype that were reported closely related to various diseases, including cancer [47]. We’ve also noticed the opposite expression patterns of FABP3 and FABP4, two fatty acid transport related genes from one family that have distinct expression pattern in different organs [48], in the Mono-like MAC from AIS/IAC or nLung. This indicated that this cluster of macrophages was regulated in a subtle way that needs further study. Immunofluorescence confirm the accumulation of FABP3 + macrophages and depletion of FABP4 + macrophages in early stage LUAD manifested as GGN (Fig. 8B). Meanwhile, genes encoding cathepsin family members (*CTSB, CTSZ* and *CTSD*), iron-metabolism associated genes (*SLC40A1, FTL*) were also upregulated. In addition, chemokines genes (*CCL18, CCL13*) were of the most upregulated genes, suggesting their potential roles in immune modulation. We also noticed that MHCII molecule including HLA-DPA1, HLA-DRB1, HLA-DQA1 and CD74 were significantly downregulated in this cluster of macrophages, which may contribute to sub-optimal tumor antigen presentation (Fig. 8A).

GSVA for Hallmark gene set (MSigDB) revealed that Mono-like Mac in AIS/IAC were enriched for many cancer-related pathways, including complement pathway, which has recently been recognized as a key component in macrophage-mediated tumor progression and immunosuppression [49]. Additionally, inflammation-related pathways and those associated with the metabolism of glucose, lipids, and iron were significantly altered. These findings collectively suggest an immune-regulated, metabolically active and protein secretion-oriented phenotype of this macrophage cluster (Fig. S15A).

Given the significant upregulation of lipid-metabolism-related genes in Mono-like Mac, we further apply GSVA using 85 previously defined gene sets related to metabolism [50]. This analysis revealed extensive alterations in metabolism, particularly in lipid-related pathways, within the Mono-like Mac cluster in AIS/IAC. These pathways encompassed processes involved in lipid synthesis, elongation, and degradation (Fig. S16A). These results further underscore the strong association of this macrophage cluster with lipid metabolism. These findings suggest that the enrichment of lipid metabolism-related macrophage clusters originating from monocytes in the early stages of lung adenocarcinoma genesis may contribute to the suppressive microenvironment and disease progression.

### Characterization of stromal cells in early-stage LUAD progression

**Fig. 9:**
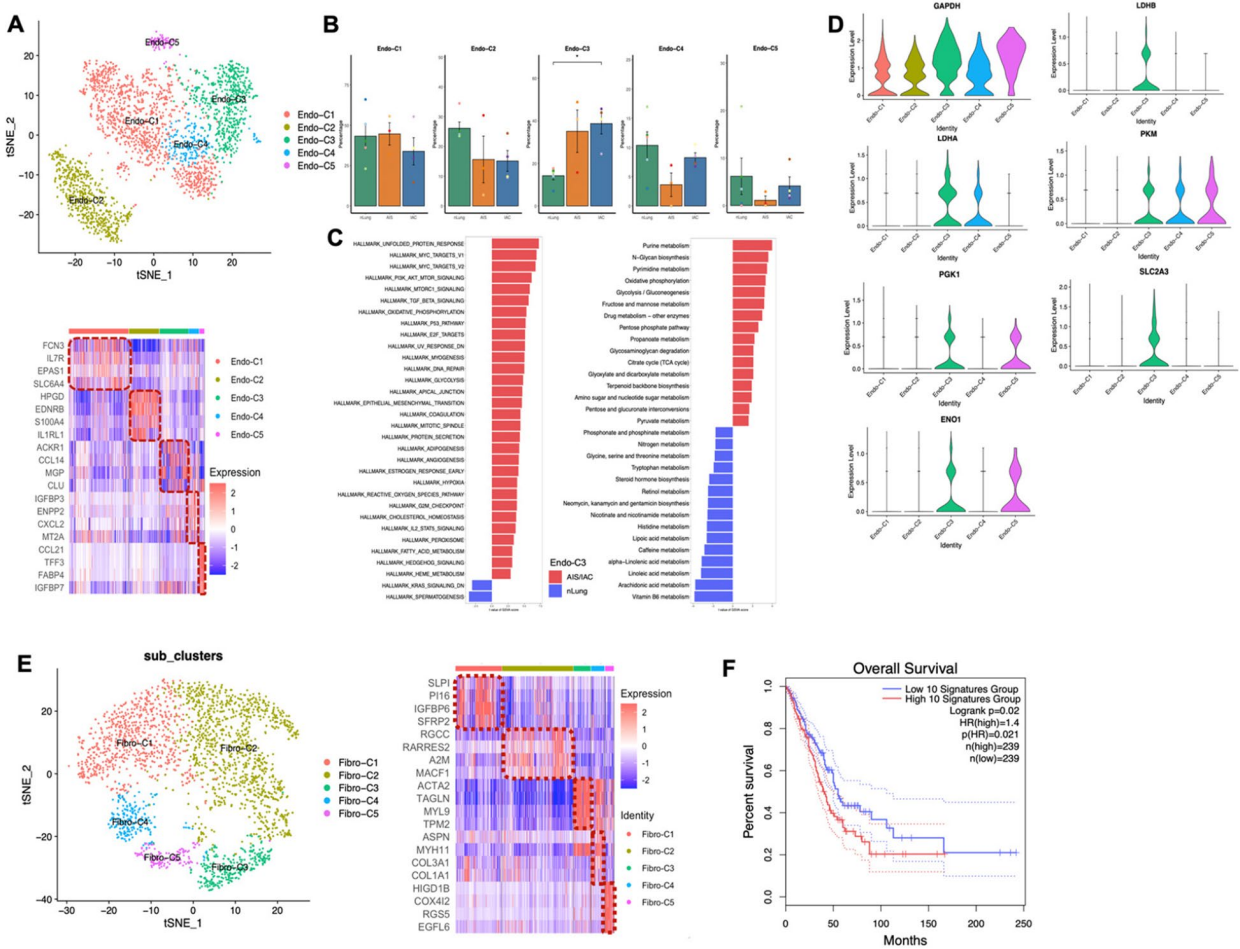
Recluster and functional analysis of stromal cells. **A** Recluster of 2,577 endothelial cells, colored according to cell subclusters (Top). Heat map showed marker genes in each subclusters (Bottom). **B** Percentage of each subclusters in different groups. Error bars represent mean ± SEM. Colored dots represent different samples. Differences with *p* < 0.05 were indicated; two-sided unpaired Wilcoxon rank sum test was used for comparison. **C** Significantly enriched Hallmark pathways in tumor-associated endothelial cells isolated from nLung or AIS/IAC as determined by GSVA score (Left). Significantly enriched metabolism-related pathways in tumor-associated endothelial cells isolated from nLung or AIS/IAC as determined by GSVA score (Right). **D** Expression of glycose-metabolism related genes in different clusters of endothelial cells. **E** Recluster of 2,513 fibroblasts, colored according to cell subclusters (Left). Heat map showed marker genes in each subclusters (Right). **F** Kaplan–Meier overall survival curves of TCGA LUAD patients with the top 10 most differentially expressed genes between fibroblasts isolated from nLung and AIS/IAC. Abbreviation: AIS: Adenocarcinoma in situ; GSVA: Gene set variation analyses; IAC: Invasive adenocarcinoma; LUAD: Lung adenocarcinoma; SEM: Standard error of mean; TCGA: The Cancer Genome Atlas Program. * *p* < 0.05

Stromal cells play a crucial role in the remodeling of the tumor microenvironment (TME) [46], yet the characteristics of stromal cells in the early progression of LUAD remain poorly understood. Therefore, next we sought to investigate single-cell transcriptomic dynamics of ECs and fibroblasts in present dataset. A total of 2,577 ECs (*RAMP2* +) were reclustered into 5 subclusters. Among these, 52.5% (1,352/2,577) originated from nLung, 28.5% (735/2,577) from AIS, and 19.0% (490/2,577) from IAC (Figs. 9A and S17A). These subclusters were characterized based on highly expressed marker genes: Endo-C1 as extra-alveolar capillary ECs (*SLC6A4* + *FCN3* + *EDN1* +), Endo-C2 as alveolar capillary ECs (*HPGD* + *EDNRB* + *IL1RL1* +), Endo-C3 as tumor-associated ECs (*PLVAP* + *SPRY1* + *HSPG2* +), Endo-C4 as arterial ECs (*GJA5* + *FBLN5* +) and Endo-C5 as lymphatic ECs (*CCL21* + *TFF3* +) (Figs. 9B and S17B). Tumor-associated ECs were enriched in IAC compared to nLung as expected (*p* = 0.028) (Fig. 9C).

Furthermore, compared to their normal counterparts, tumor-associated ECs were significantly enriched in most cancer related hallmark pathways. The top-ranking pathways including the unfolded protein response and MYC-related pathways (Fig. 9D). This observation led us to investigate the metabolic alterations in tumor-associated ECs. We found that pathways and genes related to glucose metabolism were notably altered (Fig. 9D, S18), suggesting distinct metabolic alterations in tumor-associated ECs during LUAD progression, which warrants further study.

Next, we re-clustered 2,513 fibroblasts and revealed 5 subclusters (Figs. 9E and S19A), including immune-modulatory fibroblasts (Fibro-C1; *IGFBP6* + *SFRP2* + *CCDC80* +), normal fibroblasts (Fibro-C2; *A2M* + *RGCC* +), myofibroblasts (Fibro-C3; *ACTA2* + *TAGLN* +), POSTN + fibroblast (*COL3A1* + *COL1A1* + *POSTN* +), smooth muscle cells (Fibro-C5; *ACTA2* + *COX4I2* +) (Figs. 9F and S7B). None of these clusters was significantly enriched in the AIS and IAC compared to nLung (data now shown). Direct comparison revealed that fibroblasts derived from cancer tissues were predominantly enriched for TGF-β signaling (Fig. S19C). The activation of TGF-β signaling was closely related to angiogenesis and secretion of collagens [51]. Furthermore, the most highly expressed genes in AIS/IAC-derived fibroblasts were associated with worse prognosis in LUAD dataset from TCGA (Fig. 9G).

### Ligand-receptor cell–cell communication analysis revealed immunomodulation role of stromal cells and intense interplay between myeloid cells and treg

**Fig. 10:**
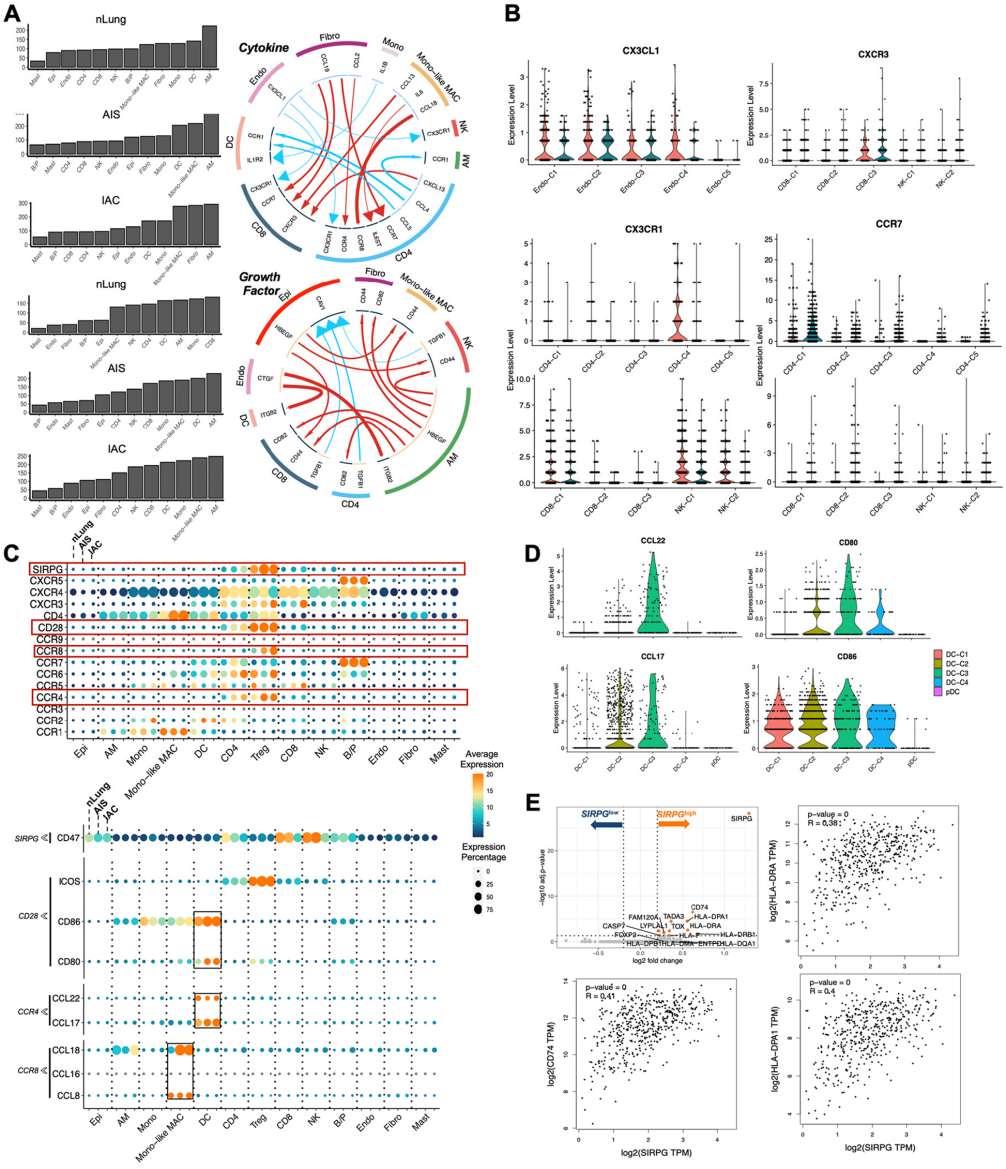
Ligand-receptor pairs-based cell–cell cross talk analysis. **A** The total number of in and out going signal from different cell types (Left). Representative circos plots showing top 15 most differentially expressed cytokine (Right Top) and growth factor (Right Bottom) L-R pairs compared between AIS/IAC and nLung. The red solid lines represent L-R pairs with higher expression in the AIS/IAC group compared to the nLung group, while the blue lines represent the opposite. The thickness of the lines represents the log2FC of ligands differential expression, and the size of the arrows represents the log2FC of receptor differential expression. The colors of the outer circle correspond to different cell types. **B** Expression pattern of CX3CL1, CX3CR1, CXCR3, and CCR7 in cell subtypes. **C** Screening of cytokine receptors on different cell types (Top). Expression of ligands for CCR4, CCR8, CD28 and SIRPG on different cell types (Bottom). **D** Expression of CCL2, CD80, CCL17 and CD86 in different clusters of DCs. **E** DEGs between Tregs with high and low expression level of SIRPG based on the mean expression level of SIRPG (Top left). Rests are the expression correlation of SIRPG with MHCII genes including HLA-DRA, CD74 and HLA-DPA1 in the LUAD cohort of TCGA. Abbreviation: AIS: Adenocarcinoma in situ; DEG: Differential expressed gene; FC: Fold of change; IAC: Invasive adenocarcinoma; LUAD: Lung Adenocarcinoma; TCGA: The Cancer Genome Atlas Program. * *p* < 0.05

The crosstalk between tumor cells, immune cells, and stromal cells in the TME forms complicated network and mediates the immunosuppressive phenotypes and tumor progression [52]. Using the iTALK R package [53], we inferred ligand-receptor cell–cell communication based on the built-in database. The total number of cell–cell interaction increased along with the disease development, indicate a more intensive signal exchange (nLung: n = 1328; AIS: n = 1580; IAC: n = 1863), and the frequency of in and out signaling in each cell clusters varied across different stages, with Mono-like MAC showing among the most altered cell types, suggesting an active phenotype (Fig. S20). Then the interactions that were significantly differed in the nLung and AIS/IAC group were analyzed and visualized separately (Fig. 10A). Firstly, we noticed a declined of CX3CL1-CX3CR1 interaction, which is reported to be an important mechanism of recruiting T and NK cells to the TME [46], between endothelium cells and T/NK cells. A detailed investigation revealed that CX3CR1 was mainly expressed by cytotoxic cell types, all of which showed decreased expression in AIS/IAC. Additionally, the expression of CX3CL1 decreased in most endothelium cells. These alterations of CX3CL1-CX3CR1 interaction may contribute to the decreased proportion of cytotoxic cells in AIS and IAC compared to nLung. Furthermore, fibroblasts exhibited intense signaling to lymphocytes through CCL19-CCR7 and CCL19-CXCR8, potentially contributing to the recruitment of naïve CD4 + T cells and ZNF683 + pre-exhausted CD8 + T cells (Fig. 10B), highlighting the immunomodulatory role of stromal cells. Additionally, HBEGF, an EGFR ligand intensively involved in cancer progression, was shown to be the main mediator of epithelial cells and AM cells affecting other cell types [54], and may serve as a potential target for early stage LUAD treatment (Fig. 10A).

As mentioned before, the accumulation of Tregs is a prominent feature of the immunosuppressive TME in initial and early-stage LUAD. To further investigate the mechanism of Treg cell recruitment, we screened the receptors expressed on Tregs. Our results showed that four receptors–CCR4, CCR8, CD28 and SIRPG–were almost exclusively highly expressed in Tregs compared with other clusters and showed trends of increased expression along with disease progression (Figs. 10C and S21A). CCR4 and CCR8 are known as important receptors for Treg recruitment and functionality [55]. Screening for ligands revealed that Mono-like MAC and DCs have the most interactions with Treg through CCL18/CCL8-CCR8, CCL17/CCL22-CCR4 and CD80/86-CD28, respectively (Figs. 10C and S21B). In detail, DC-C3 (LAMP3 + DC) cluster was the major source of CCL22, CCL17 and CD80, which is consistent with recent studies highlighting the role of LAMP3 + DCs in creating immunosuppressive environment (Fig. 10D) [56]. These findings suggest that recruitment into TME by myeloid cells may serve as a third source of Treg accumulation in the TME of initial and early-stage LUAD, in addition to local expansion and differentiation from naïve CD4 + T cells as previously mentioned in this study.

Unlike SIRPA, which has been extensively studied [57], very few studies have focused on the role of SIRPG in the immune system and Treg regulation. Direct comparison of Tregs that have high or low expression of SIRPG unexpectedly revealed an upregulation of MHC II molecules, including CD74, HLA-DPA1, HLA-DRA, HLA-DRB1. SIRPG was also positively related with CD74, HLA-DPA1, HLA-DRA in the LUAD dataset from TCGA (Fig. S22A). This suggests that SIRPG may serve as a novel marker for the MHC II expressed Tregs which was highly active and immunosuppressive [58], presenting a potential new target for eliminating Tregs in very early-stage LUAD. In addition, CD47, the known gland for SIRPG, showed ubiquitously expression in all cell clusters, suggesting extensive interactions of Tregs with other cellular components through the CD47-SIRPG pathway. The mechanisms underlying these interactions warrant further investigation.

## Discussion

The molecular and immunological mechanism underlying the genesis and progression of early-stage LUAD remain elusive. In this study, we depict the dynamic molecular and cellular landscape of the initial stages of LUAD through single-cell RNA/TCR integrated analysis. We have revealed the dynamic molecular and cellular trajectory during malignant progression of LUAD manifested as GGN. These findings which may lay foundation for the development of novel therapeutic and diagnostic targets.

By pre-filtering genes with disease stage-related expression patterns and combining single-cell WGCNA, we have delineated five signatures that over-represent the disease-stage related mechanism that contributing to malignant progression from normal lung tissue to AIS and early-stage IAC. Pathways associated with metabolism, proliferation and signal transduction were continuously increased, which is consistent with the sustained proliferative nature of cancer cells [59]. Additionally, cell death regulation pathways were also assigned to the continuously ascending module, suggesting the early raised and sustained disturbance of balance between the immune surveillance and anti-apoptotic nature of cancerous cells. We also observed an acute upregulation of antigen presentation and cytokine pathway responses from the stage of AIS, reflecting active immunological interactions between abnormal epithelial cells and the host immune system. This suggested that pre-cancerous lung epithelial cells possess immunogenicity and engage in intense interactions with the immune system. These findings align with previous studies indicating that the adaptive immune response is strongest at the earliest stages of carcinoma [60, 61]. This may partly explain the indolent growth pattern observed in GGNs.

We also noticed that pathways associated with mitochondrial organization and function displayed a biphasic pattern and reached their peak in the AIS stage. This observation aligns with previous research in skin tumors revealed that mitochondrial respiration strengthens before tumor establishment, and targeting electron transport chain (ETC) could inhibit the formation of skin tumors [62]. Similarly, in a mouse model of Kras-induced pancreatic cancer [63], Humpton et.al reported that Nix-mediated mitophagy occurred during the stage of pancreatic intraepithelial neoplasia (PanIN), the pre-cancerous lesion of pancreatic cancer, to drive redox robustness and disease progression. Based on our data and previous research, we propose a model in which mitochondria and oxidative phosphorylation may serve as the primary source of energy production at the very beginning of LUAD tumorigenesis. Subsequently, energy metabolism undergoes reprogramming as the disease progresses. Further investigations into the mechanisms underlying this reprogramming and the downregulation of mitochondria may offer insights into novel therapeutic targets and metabolic imaging tools for early-stage LUAD [64]. Another biphasic signature we observed was closely related with cilia, although we cannot determine whether pertains to primary cilia or motile cilia. Primary cilia were considered to be organelle with cancer suppression function [65], on the other hand, lung motile cilia were reported to involve in the oxidative stress regulation [66], which can also be dysregulated in cancer development, the biological consequences of cilia disorder will worth further exploration since lung is motile cilia enrich tissue. In addition, continuously decrease in cell adhesion and cell junction -associated pathways suggests the acquisition of invasiveness. In summary, our data provide insights into how lung epithelial cells progress to malignant cells from various perspectives.

Immunotherapy such as PD-1/PD-L1 inhibitors holds great promise as therapeutic approach for various cancers, however, current evidence [67], albeit still preliminary, suggests a limited effect of immunotherapy on early-stage lung cancer manifested as GGN. The underlying mechanism underlying are still elusive. In the current study, we observed that the immune microenvironment in early-stage LUAD is primarily characterized by several key features. These include the exclusion of NK cells, the accumulation of pre-exhausted CD8+ T cells, and a reduction in the effector function of T cells. Notably, we did not identify clusters of exhausted CD8+ T cells highly expressing PD-1 and other co-inhibitory molecules in pre-cancerous lesions and early-stage LUAD. We believe that these findings could partially account for the limited effectiveness of PD-1 inhibitors in this particular subset of patients. Indeed, the CD8+ T cell landscape in GGN closely resembles the characteristics observed in late-stage LUAD following responsiveness to PD-1 inhibitor treatment, that is the accumulation of pre-exhausted cells with dramatically decreased of terminal exhausted CD8+ T cells [20]. This similarity may reflect immune competence of AIS and early stage LUAD manifested as GGN, which is also consistent with the indolent growth pattern of GGN in the clinical observation. The dysfunction of CD8+ T cells in AIS and early-stage LUAD is associated with decreased effector function and a shift toward a memory phenotype. We have identified transcriptional programs associated with these changes, and some molecules may serve as potential targets for reinvigorating of T cells in early stage LUAD patients. For instance, PCBP1, a newly defined checkpoint for T cell function recently, is a promising candidate [38]. TCR analysis has revealed a decreased clonal cell ratio of T cells, except for Treg cells. This also suggests a compromised proliferation capacity of T cells in response to carcinogenesis, which is consistent with the observed decrease in effector function. Furthermore, the analysis of TCR overlapping, combined with trajectory analysis, has shown that the transition of CX3CR1+ cytotoxic CD8+ T cells into GZMK+ pre-exhausted CD8+ T cells may be a crucial mechanism underlying the loss of CD8+ cell function. This finding is reminiscent of a previous study proposing that exhausted CD8+ T cells in the TME of nasopharyngeal carcinoma may originate from peripheral CX3CR1+CD8+ T cells [68]. Studies have also suggested that pre-exhausted CD8+ T cells are precursors of terminal exhausted T cells [23]. The mechanism that lead to CD8+ T cells persisting in the pre-exhausted status in GGN, such as low antigen loads due to low tumor mutation burden (TMB) [11], or other factors, require further investigation. Furthermore, the decreased interaction between endothelial cells and cytotoxic cell clusters through CX3CL1-CX3CR1 may impair the chemotaxis of cytotoxic cell clusters, including NK and CD8+ T cells, into the TME. This deficit could further contribute to the loss of functional status in the early progression of LUAD.

The enrichment of Tregs is another characteristic feature observed in pre-cancerous and early-stage LUAD. We have provided evidence for three potential sources of Treg accumulation in this context: local expansion, differentiation from naïve CD4+ T cells, and recruiting by other cell types through cytokine and receptor interaction. Notably, myeloid cells, including monocyte-derived macrophages and LAMP3+ DCs, appear to play a significant role in recruiting Tregs. To effectively target Tregs with different sources, it may be necessary to employ a combination of different approaches. We have also identified receptors that are preferentially expressed on Tregs, including CCR4, CCR8, CD28 and SIRPG. CCR4 and CCR8 have been extensively studied as potential targets for elimination Tregs in the TME [55, 69], and our data supports the possibility of implement CCR4 and CCR8 inhibitors in GGN patients in the future. In contrast, the role of SIRPG in Tregs has been less explored, and it has often been considered less functional than SIRPA [70], another member from the same family. Interestingly, we found for the first time that SIRPG can be a marker of MHCII+ Tregs in early-stage LUAD. MHCII+ Tregs are considered mature Treg effector cells [71] with high contact-dependent suppressive capacity [72]. The ubiquitous expression of its ligand, CD47, also suggests that SIRPG+ Treg can form extensive contacts with other cellular components. This discovery may provide new insights into the development of Treg-targeted therapies.

The role of macrophages in cancer has been widely studied, primarily in the context of metastasis and resistance to treatment [73]. However, our study has identified a cluster of monocyte-derived and lipid-associated macrophages that may play an important role in the early stages of carcinogenesis. This is based on their accumulation in precancerous and early-stage LUAD, as well as their high expression of pro-tumorigenic molecules such as CCL18, CSTs, and GPNMB. Recent study reported that alveolar resident macrophages can accumulate in LUAD genesis and exert pro-tumorigenic function in the very beginning of tumorigenesis in mouse models [74]. As complement, we showed that monocytes derived macrophages may also play pro-tumorigenic roles in the very early stage of LUAD. Except for macrophages, we also highlighted that LAMP3+ DCs may represent another type of highly active myeloid cells with intense crosstalk with Tregs. LAMP3+ DCs has drawn much attention in recent years as an emerging key player in suppressive TME, and have been observed in various types of cancers [56], which holds potential as novel targets for immunotherapy.

There are also several limitations of our study, first, the sample size in our study was relatively small. Second, we are unable to sample the same lesion at different timepoints to conduct longitudinal study. In the future, our findings can be further validated in larger cohorts, or with the help of patient-derived xenografts or genetically engineered spontaneous LUAD mouse model that can mimic the different phase of LUAD genesis [75]. New technologies, such as single-cell spatial transcriptomics, may also help deepen our understanding of the complex cellular and molecular networks involved in the genesis and progression of early-stage LUAD.

## Conclusions

In conclusion, through single-cell RNA and TCR combined sequencing and analysis, we have delineated the molecular and cellular trajectory during the malignant progression of LUAD manifested as GGN. This provides new insight into the mechanisms underlying the indolent grow pattern of GGN and unveils key features that dominant the TME of GGN, along with the mechanisms that may promote the progression from precancerous lesions to LUAD. We believe that our findings could pave way for novel immunotherapies for GGN, which is currently in severe lack of internal medicine pharmacotherapy.

### Supplementary Information

**Additional file 1:** **Fig S1. **A. TSNE plot of 38,814 cells, colored according to all the cell subtypes and split by the origin of the cells respectively according to origin of the cells. Each dot represents a single cell. B. TSNE plot of 1,183 normal epithelial cells from nLung, colored according to cell subtypes (Top left), colored according to number of genes detected (Top right), colored according to the origin of the cells (Bottom left) and canonical epithelial markers expression across subtypes (Bottom right). C. Heat map showed marker genes in each subclusters. Abbreviation:TSNE: T-distributed Stochastic Neighbor Embedding. **Fig S2. **Top 25 genes with maximum connectivity with other genes (based on module eigengene-base connectivity) in five modules derived from scWGCNA analysis. Abbreviation:scWGCNA:Single-Cell Weighted Gene Co-expression Network Analysis.** Fig S3. **A. TSNE plot of 11,847 T/NK cells, colored according to the number of genes detected (Top left), colored according to the origin of the cells (Top right), and split by the origin of the cells respectively according to origin of the cells (Bottom). Each dot represents a single cell. Abbreviation: TSNE: T-distributed Stochastic Neighbor Embedding. **Fig S4. **A. TSNE plot of 9,179 myeloid cells, colored according to the number of genes detected (Top left), colored according to the origin of the cells (Top right), and split by the origin of the cells respectively according to origin of the cells (Bottom). Each dot represents a single cell. B. Canonical markers expression for each myeloid subclusters. Abbreviation:TSNE: T-distributed Stochastic Neighbor Embedding. **Fig S5. **A. Expression of functional markers for each myeloid subclusters. **Fig S6. **A. TSNE plot of 2,577 endothelial cells, colored according to the number of genes detected (Top left), colored according to the origin of the cells (Top right), and split by the origin of the cells respectively according to origin of the cells (Bottom). Each dot represents a single cell. B. Canonical markers expression for each endothelial subclusters. Abbreviation: TSNE: T-distributed Stochastic Neighbor Embedding. **Fig S7. **A. TSNE plot of 2,513 fibroblasts, colored according to the number of genes detected (Top left), colored according to the origin of the cells (Top right), and split by the origin of the cells respectively according to origin of the cells (Bottom). Each dot represents a single cell. B. Canonical markers expression for each fibroblast subclusters. C. Significantly enriched Hallmark pathways in Fibro-C4 isolated from nLung or AIS/IAC as determined by GSVA score. Abbreviation: AIS: Adenocarcinoma in situ; GSVA: Gene set variation analysis; IAC: Invasive adenocarcinoma; nLung: normal lung; TSNE: T-distributed Stochastic Neighbor Embedding. **Fig S8. **A. Cytokine receptors expression patters in each cell clusters across different groups. B. Cytokine ligands expression patterns in each cell clusters across different groups.

## Data Availability

All reagents and experimental protocols described in this work are available upon reasonable request. Correspondence and requests for materials should be addressed to ZQW and XJZ.

## Abbreviations

LUAD: Lung adenocarcinoma
TCR: T cell receptor
TME: Tumor micro-environment
Tregs: Regulatory T cells
GGN: Ground glass nodule
NLST: National Lung Screening Trial
CXR: Chest radiography
LDCT: Low Dose Computed Tomography
AAH: Atypical Adenomatous Hyperplasia
IAC: Invasive Adenocarcinoma
scRNA: Single-Cell RNA
DEG: Differential Expressed Genes
GO: Gene Ontology
GSVA: Gene Set Variation Analyses
FDR: False-Discovery Rate
SCIENIC: Single-Cell Regulatory Network Inference and Clustering
ETC: Electron Transport Chain
TMB: Tumor Mutation Burden
AIS: Adenocarcinoma In Situ
MIA: Microinvasive Adenocarcinoma
