# Supplementary material for: Comparative immunological landscape between pre- and early-stage LUAD manifested as ground-glass nodules revealed by scRNA and scTCR integrated analysis
Source: Cell Commun Signal. 2023 Nov 13;21:325. doi: 10.1186/s12964-023-01322-x (PMC10644515; doi:10.1186/s12964-023-01322-x)
Supplement: Supplementary file 2 — Additional file 1: Fig S1. A. TSNE plot of 38,814 cells, colored according to all the cell subtypes and split by the origin of the cells respectively according to origin of the cells. Each dot represents a single cell. B. TSNE plot of 1,183 normal epithelial cells from nLung, colored according to cell subtypes (Top left), colored according to number of genes detected (Top right), colored according to the origin of the cells (Bottom left) and canonical epithelial markers expression across subtypes (Bottom right). C. Heat map showed marker genes in each subclusters. Abbreviation:TSNE: T-distributed Stochastic Neighbor Embedding. Fig S2. Top 25 genes with maximum connectivity with other genes (based on module eigengene-base connectivity) in five modules derived from scWGCNA analysis. Abbreviation:scWGCNA:Single-Cell Weighted Gene Co-expression Network Analysis. Fig S3. A. TSNE plot of 11,847 T/NK cells, colored according to the number of genes detected (Top left), colored according to the origin of the cells (Top right), and split by the origin of the cells respectively according to origin of the cells (Bottom). Each dot represents a single cell. Abbreviation: TSNE: T-distributed Stochastic Neighbor Embedding. Fig S4. A. TSNE plot of 9,179 myeloid cells, colored according to the number of genes detected (Top left), colored according to the origin of the cells (Top right), and split by the origin of the cells respectively according to origin of the cells (Bottom). Each dot represents a single cell. B. Canonical markers expression for each myeloid subclusters. Abbreviation:TSNE: T-distributed Stochastic Neighbor Embedding. Fig S5. A. Expression of functional markers for each myeloid subclusters. Fig S6. A. TSNE plot of 2,577 endothelial cells, colored according to the number of genes detected (Top left), colored according to the origin of the cells (Top right), and split by the origin of the cells respectively according to origin of the cells (Bottom). Each dot represent [file 12964_2023_1322_MOESM1_ESM.docx]

**Supplementary**

**
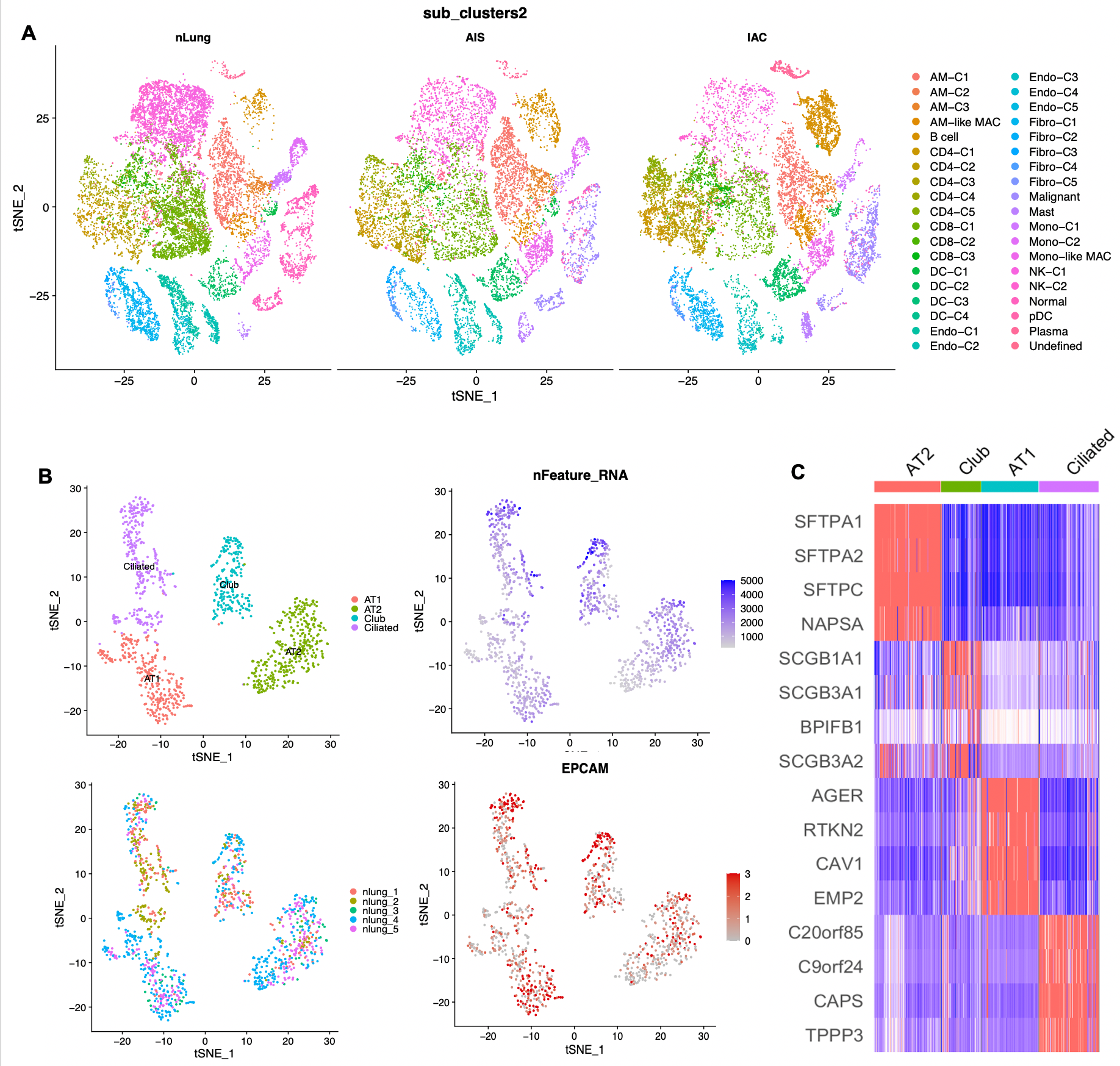
**

**Fig S1.** A. TSNE plot of 38,814 cells, colored according to all the cell subtypes and split by the origin of the cells respectively according to origin of the cells. Each dot represents a single cell. B. TSNE plot of 1,183 normal epithelial cells from nLung, colored according to cell subtypes (Top left), colored according to number of genes detected (Top right), colored according to the origin of the cells (Bottom left) and canonical epithelial markers expression across subtypes (Bottom right). C. Heat map showed marker genes in each subclusters. Abbreviation: **TSNE**: T-distributed Stochastic Neighbor Embedding.

**
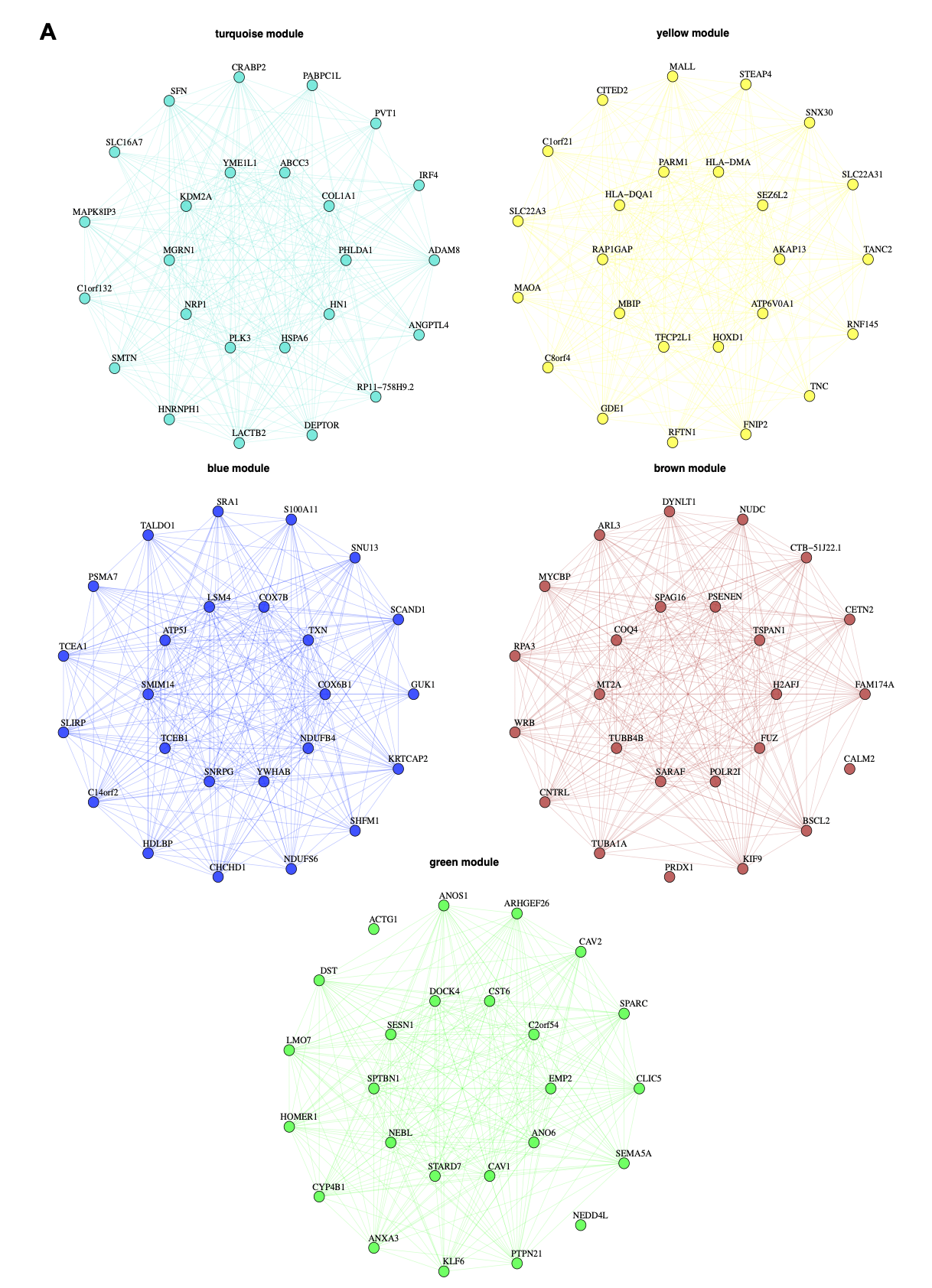
**

**Fig S2.** Top 25 genes with maximum connectivity with other genes (based on module eigengene-base connectivity) in five modules derived from scWGCNA analysis. Abbreviation: **scWGCNA:** Single-Cell Weighted Gene Co-expression Network Analysis.

**
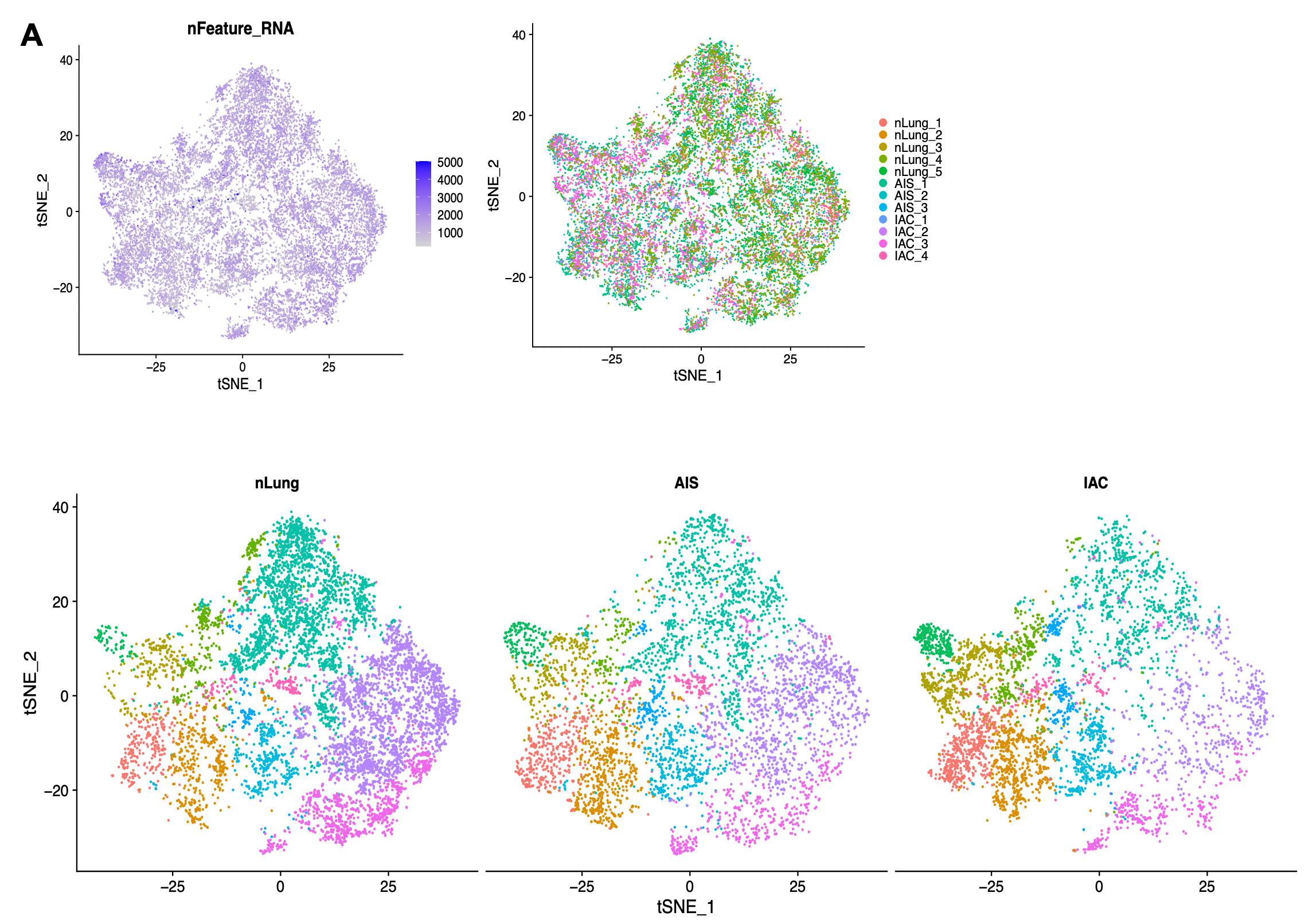
**

**Fig S3.** A. TSNE plot of 11,847 T/NK cells, colored according to the number of genes detected (Top left), colored according to the origin of the cells (Top right), and split by the origin of the cells respectively according to origin of the cells (Bottom). Each dot represents a single cell. Abbreviation: **TSNE**: T-distributed Stochastic Neighbor Embedding.

**
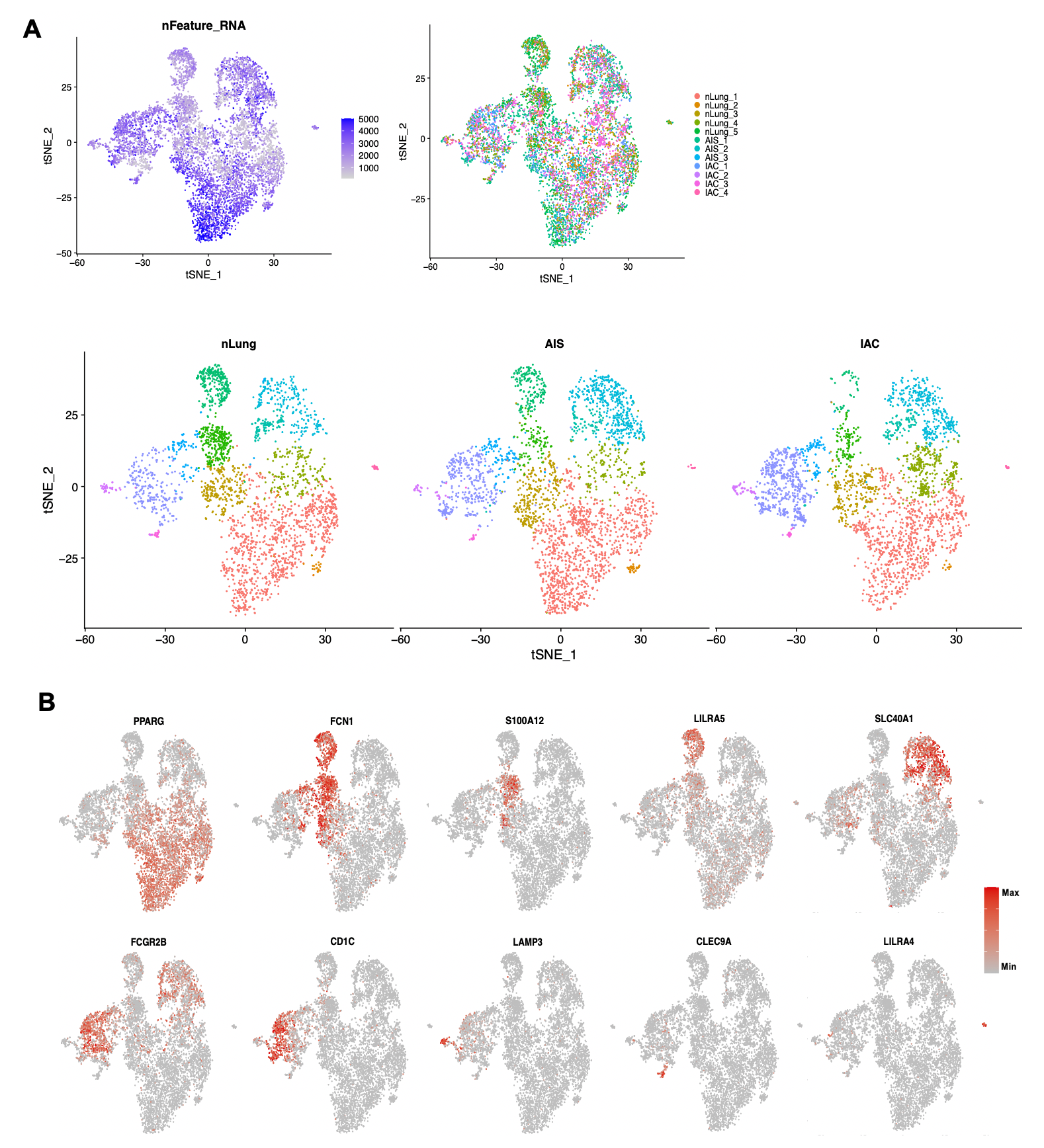
**

**Fig S4.** A. TSNE plot of 9,179 myeloid cells, colored according to the number of genes detected (Top left), colored according to the origin of the cells (Top right), and split by the origin of the cells respectively according to origin of the cells (Bottom). Each dot represents a single cell. B. Canonical markers expression for each myeloid subclusters. Abbreviation: **TSNE**: T-distributed Stochastic Neighbor Embedding.

**
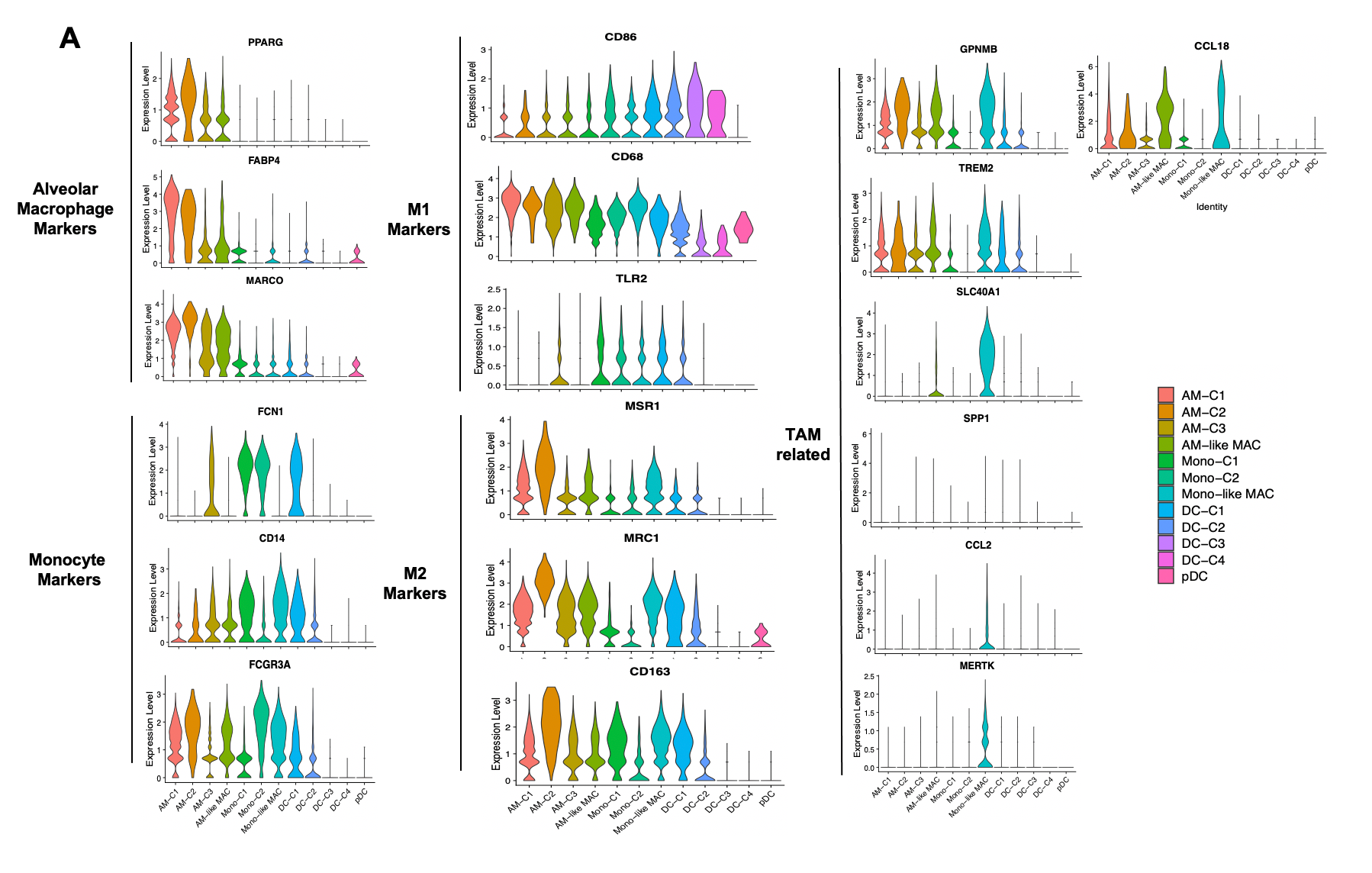
**

**Fig S5.**A. Expression of functional markers for each myeloid subclusters.

**
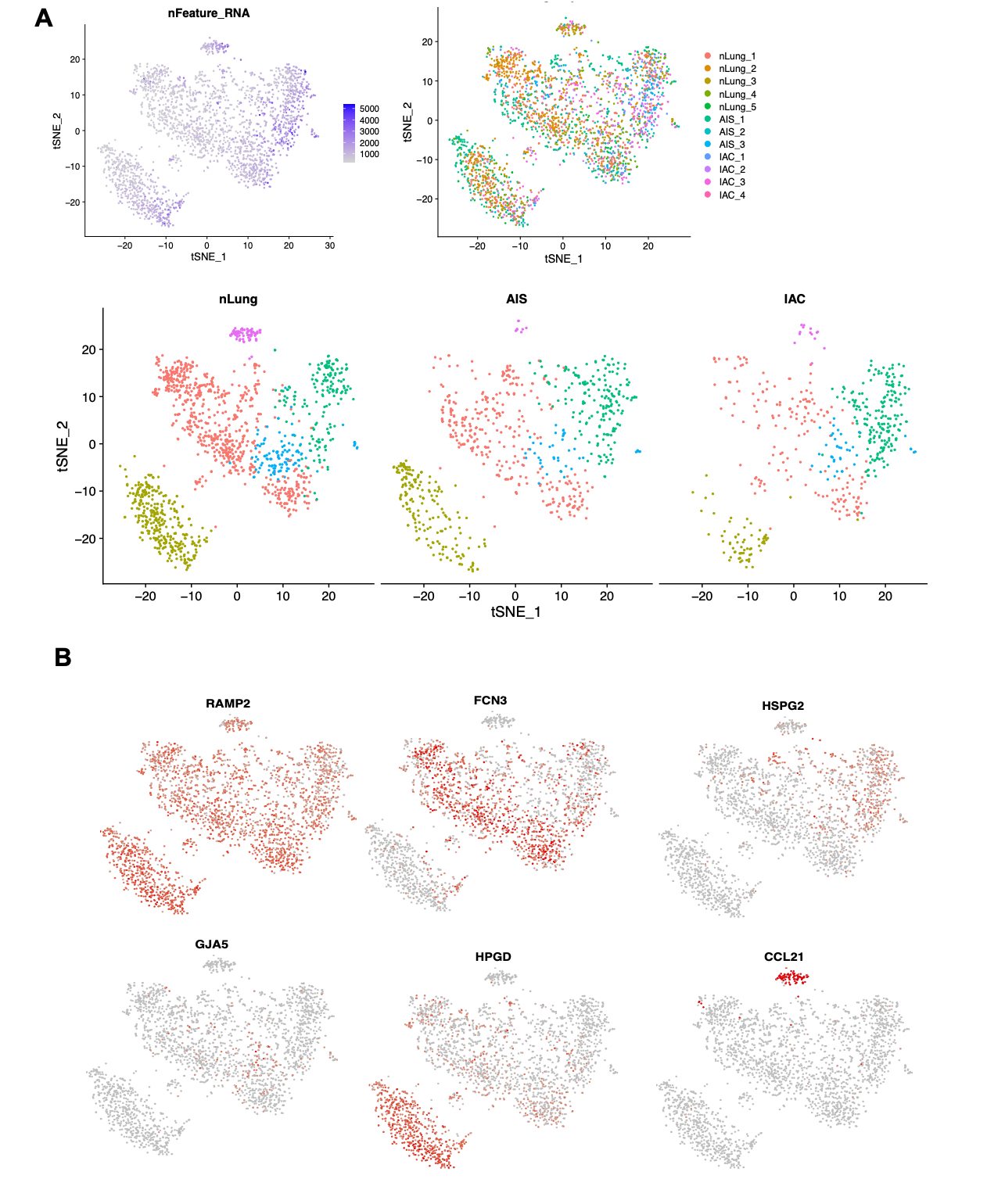
**

**Fig S6.** A. TSNE plot of 2,577 endothelial cells, colored according to the number of genes detected (Top left), colored according to the origin of the cells (Top right), and split by the origin of the cells respectively according to origin of the cells (Bottom). Each dot represents a single cell. B. Canonical markers expression for each endothelial subclusters. Abbreviation: **TSNE**: T-distributed Stochastic Neighbor Embedding.**
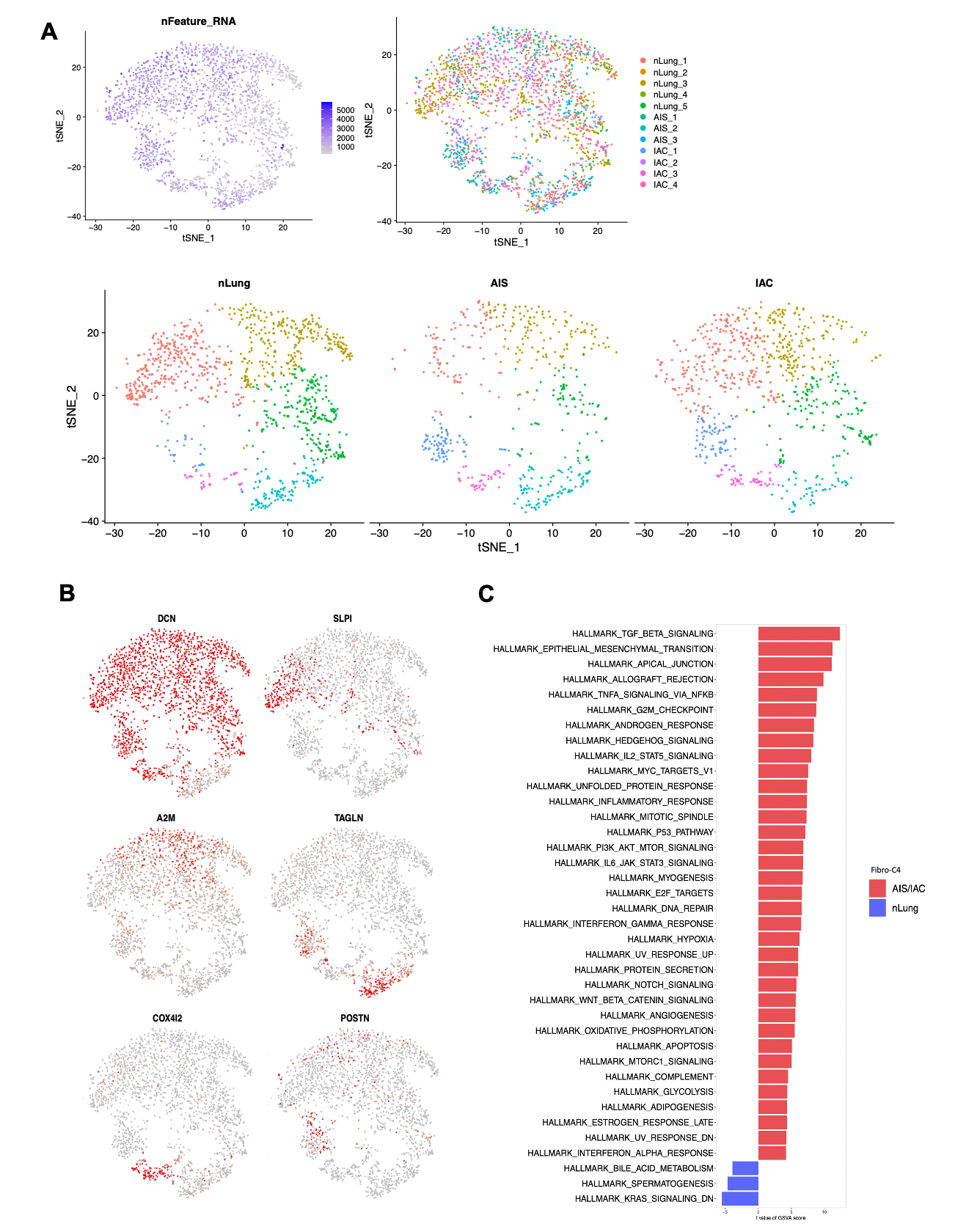
**

**Fig S7.** A. TSNE plot of 2,513 fibroblasts, colored according to the number of genes detected (Top left), colored according to the origin of the cells (Top right), and split by the origin of the cells respectively according to origin of the cells (Bottom). Each dot represents a single cell. B. Canonical markers expression for each fibroblast subclusters. C. Significantly enriched Hallmark pathways in Fibro-C4 isolated from nLung or AIS/IAC as determined by GSVA score. Abbreviation: **AIS**: Adenocarcinoma in situ; **GSVA**: Gene set variation analysis; **IAC**: Invasive adenocarcinoma; **nLung:** normal lung; **TSNE**: T-distributed Stochastic Neighbor Embedding.

**
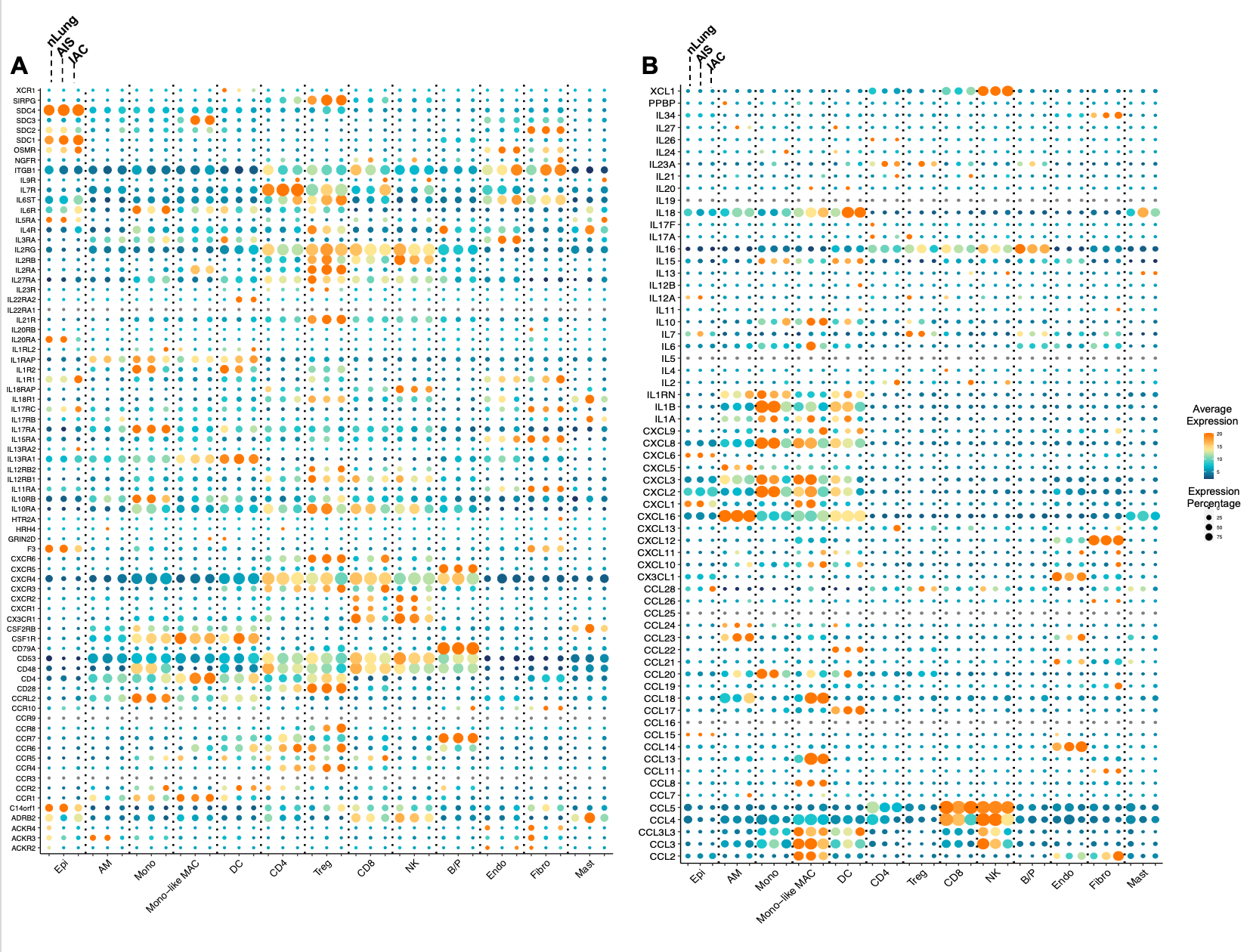
**

**Fig S8.** A. Cytokine receptors expression patters in each cell clusters across different groups. B. Cytokine ligands expression patterns in each cell clusters across different groups.
